# Proteomics Approach to Differentiate Protein Extraction Methods in Sugar Beet Leaves

**DOI:** 10.1021/acs.jafc.2c09190

**Published:** 2023-06-05

**Authors:** Ece Goktayoglu, Mecit Halil Oztop, Sureyya Ozcan

**Affiliations:** † Department of Food Engineering, Middle East Technical University, 06800 Ankara, Turkiye; ‡ Department of Chemistry, Middle East Technical University, 06800 Ankara, Turkiye; ¶ Department of Chemical Engineering, University of California Davis, Davis, California 95616, United States

**Keywords:** plant-based protein, sugar beet leaves, *Beta vulgaris*, mass spectrometry, proteomics, RuBisCO protein

## Abstract

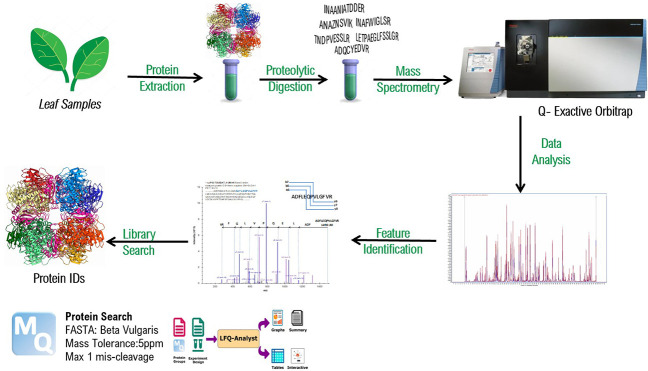

Interest in alternative plant-based protein sources is continuously growing. Sugar beet leaves have the potential to satisfy that demand due to their high protein content. They are considered as agricultural waste and utilizing them as protein sources can bring them back to the food chain. In this study, isoelectric-point-precipitation, heat-coagulation, ammonium-sulfate precipitation, high-pressure-assisted isoelectric-point precipitation, and high-pressure-assisted heat coagulation methods were used to extract proteins from sugar beet leaves. A mass spectrometry-based proteomic approach was used for comprehensive protein characterization. The analyses yielded 817 proteins, the most comprehensive protein profile on sugar beet leaves to date. Although the total protein contents were comparable, there was a significant difference between the methods for low-abundance proteins. High-pressure-assisted methods showed elevated levels of proteins predominantly located in the chloroplast. Here we showed for the first time that the extraction/precipitation methods may result in different protein profiles that potentially affect the physical and nutritional properties of functional products.

## Introduction

In today’s world, the interest in plant-based proteins has increased considerably. Production and consumption of plant-based proteins is considered more sustainable since animal-based production is known to cause 75% of total agricultural emissions.^1^ Therefore, there is an increasing demand for plant based proteins. To this end, the production of alternative protein sources from plants are continuously explored.

Sugar beet (*Beta vulgaris L.*) is a flowering plant that belongs to the Amaranthaceae family, and its taxonomical position is in the order Caryophyllales.^2^ Sugar beet and sugar cane are the two important sources of white sugar that are consumed worldwide. Sugar beet accounts for 20% of the world sugar production, and the rest is obtained from cane.^3^ Turkey, Poland, Ukraine, and Russia are the main beet sugar producers in the world.^4^ In the course of beet sugar production, the roots are harvested, and leaves remain on the field and are not utilized efficiently. They are often used as animal feed, but a huge fraction remains in the field; thus, they are considered as *agricultural waste*.^5^ Since beet leaves constitute approximately 30% of the plant,^6^ their valorization is significant. The leaves contain mainly 18% cellulose, 17% hemicellulose, 6% lignin, and 6% pectin in dry basis.^7^ They also contain a high amount of crude protein, between 18 and 23% on a dry basis.^6,8^ In addition, leaves contain some of the essential amino acids such as leucine, valine, phenylalanine, lysine, threonine, isoleucine, and methionine,^5,9^ which contribute to nutritional quality of the leaf. With the presence of these amino acids, sugar beet leaves can be considered as nutritionally complete protein and have the potential to be an alternative protein source like soy protein.^10^ Therefore, obtaining proteins from the leaves is important from both valorization and sustainability perspectives.

More than 50% of the leaf proteins are soluble proteins that are located in the stroma,^6,11^ and 70% of the leaf proteins are in the chloroplast;^12^ including insoluble proteins in the thylakoid membrane.^11^ The predominant protein in leaves (approximately 50% of total soluble proteins) is ribulose 1,5-biphosphate carboxylase/oxygenase (RuBisCO), which is responsible for the fixation of CO_2_ during photosynthesis.^6,13^ RuBisCO is also the most abundant protein in nature.^14^

Proteins in leaves are embedded in the cellulosic matrix. By applying various extraction methods, proteins can be purified from leaves and enriched for further use. Proteins are basically separated from any extra contaminants, by decreasing their solubility. Sugar beet leaf proteins are extracted using isoelectric point, heat coagulation, or salt precipitation.^5,6^ Plant-based protein isolation methods are mostly induced by protein precipitation. The process involves protein denaturation that also assists proteolytic digestion for protein identification. Although denatured proteins lose their biological function, they maintain the nutritional value. The main purpose in the extraction process is to disrupt the cells containing the protein.^15,16^ Due to the presence of a strong cellulosic matrix and lignin, extracting proteins with a yield above 80% has been a challenge. Thus, different strategies have been proposed to increase the extraction efficiency. Depending on where the proteins are located and how fragile they are, the methods vary including *ultrasonication* (US), *high-pressure homogenization* (HHP), and *pulsed electric field* (PEF).

Extraction and physical characterization of sugar beet leaf proteins by different techniques have been studied.^5,6^ However, the comparison of the methods were only on crude/total protein level. The nutritional and physical properties of protein extracts are highly dependent on the protein composition. Therefore, there is a need for comprehensive protein analysis for protein extracts isolated from sugar beet leaf. By using mass spectrometry-based proteomics, it is possible to make both qualitative and quantitative analysis of a variety of proteins even in complex systems. Two proteomics studies were previously reported on sugar beet leaves; however they investigated the applied drought stresses^17^ and *rhizomania*, (one of the most damaging diseases of sugar beet), effects on the proteins.^18^ The different protein enrichment methods that have been used in these studies showed that different proteins exist in the sugar beet leaves in varying amounts so, the data obtained in this study will be a reference for the use of sugar beet leaves as an alternative plant-based protein source.

In this study, mass spectrometry-based proteomic analysis of sugar beet leaf extracts obtained by different techniques, isoelectric point precipitation, heat coagulation, ammonium sulfate precipitation, and high-pressure-assisted isoelectric point precipitation and heat coagulation, have been investigated. Proteomics data have been included as a comprehensive analysis for the protein from beet leaves.

## Methods

### Materials

Sugar beet leaves, harvested during fall 2021 were obtained from Kayseri Şeker Co. (Kayseri, Turkiye). Sodium bicarbonate (NaHCO_3_), sodium carbonate (Na_2_CO_3_), hydrochloric acid (HCl), sodium hydroxide (NaOH), ammonium sulfate ((NH_4_)_2_SO_4_), and formic acid (HCOOH) were purchased from Sigma-Aldrich Chemical Co. (St. Louis, MO, USA). Filter-assisted sample preparation (FASP) digestion kits were purchased from Expedeon Inc. (San Diego, CA, USA).

### Sample Preparation

Fresh leaves were homogenized with a Thermomix (Vorwerk, Turkiye) at 6000 rpm for 2 min right after delivery and stored in plastic bags at −20 °C for further analysis. Since the bags contained a mix of the homogenized leaves and they were all stored in homogenized form effect of “different water content” has been minimized to a great extent.

### Protein Isolation

Sugar beet leaf proteins were extracted using three different precipitation methods, and to increase the extraction yield, high pressure was applied in some methods: (1) isoelectric point precipitation, (2) heat coagulation, (3) ammonium sulfate precipitation, (4) high-pressure-assisted isoelectric point precipitation, and (5) high-pressure-assisted heat coagulation. Extractions were completed as follows.

#### Protein Extraction with Isoelectric Point Precipitation

The leaves were thawed at room temperature and then mixed with pH 9.2, 0.02 M carbonate–bicarbonate buffer at a 1:8 (w/v) ratio and homogenized using IKA T18 digital Ultra-Turrax (Germany) at 10000 rpm for 4 min. The homogenized solution was then heated up to 50 °C and shaken at 80 rpm for 30 min in a water bath. After filtration, the solution was centrifuged at 6000 rpm for 20 min with NF 1200R Bench-Top Cooled Centrifuge (Turkiye). For isoelectric point precipitation, the pH of the supernatant was adjusted to 3.5 with 3 N HCl, and the solution was stirred with a magnetic stirrer (Daihan Scientific Co., Ltd., Korea) at 300 rpm for 30 min. The suspension was then centrifuged at 6000 rpm for 30 min, and the pellet was collected. Finally, the obtained pellet was freeze-dried for 24 h (Beijing Songyuan Huaxing Technology Development Co., Ltd., China). Protein powders were stored at 4 °C after lyophilization for further analysis.

#### Protein Extraction with Heat Coagulation

This method was developed by adding an additional step to the isoelectric point precipitation method. Following the first centrifugation, the suspension was heated up to 80 °C and shaken at 80 rpm for 30 min in a water bath. Later, the pH of the suspension was adjusted to 3.5 by using 3 N HCl, and the suspension was continuously stirred. Further steps were same as the isoelectric point precipitation.

#### Protein Extraction with Ammonium Sulfate Precipitation

In this method, one step of isoelectric point precipitation method was modified only. After centrifugation, the supernatant was collected, and ammonium sulfate was added at a concentration of 85% (w/v) to the supernatant. The suspension was stirred with a magnetic stirrer at 300 rpm for 60 min. The proteins were collected via centrifugation at 6000 rpm after 30 min. The excess ammonium sulfate from the pellet was removed by dialysis at room temperature for 24 h. Finally, the solution was freeze-dried and obtained protein powders were stored at 4 °C for further experiments.

#### Protein Extraction with High-Pressure-Assisted Isoelectric Point Precipitation

The extraction method was applied by the same procedure as isoelectric point precipitation with only one adjustment. After the leaves were mixed with the buffer solution, the suspension was homogenized and shaken for 30 min at 80 rpm and 50 °C. Later, the suspension was passed through the GEA PandaPLUS Lab Homogenizer 2000 (GEA Mechanical Equipment IT S.p.A, Parma, Italy) for further extraction. The mixture was exposed to 3 passes at 1400 bar. The rest of the process was carried out as described in the previous method.

#### Protein Extraction with High-Pressure-Assisted Heat Coagulation

The extraction method was applied by the same procedure as heat coagulation with only one adjustment. After the leaves were mixed with the buffer solution, the suspension was homogenized and shaken for 30 min at 80 rpm and 50 °C. Later, the suspension was passed through the GEA PandaPLUS Lab Homogenizer 2000 (GEA Mechanical Equipment IT S.p.A, Parma, Italy) for further extraction. The mixture was exposed to 3 passes at 1400 bar. The rest of the process was carried out as described in the previous method.

### Proteomics Sample Preparation

Further protein extraction was assisted by using UPX buffer and Filter-Assisted Sample Preparation digestion kit (Expedeon Inc., San Diego, CA, USA). In this kit, sodium dodecyl sulfate (SDS) was used as alkalizing agent, and proteins were digested with trypsin. Obtained peptides were suspended in 0.1% formic acid and stored at −80 °C prior to the MS analysis. For each method, two biological and two technical replicates were analyzed.

### MS Analysis

The peptides were separated using an UltimateTM 3000 RSLC Nano Ultra-Liquid Chromatography system (Thermo Scientific) coupled with a Q-Exactive HF-X Mass Spectrometer (Thermo Fisher Scientific, Bremen, Germany) through an EASY-Spray TM (Thermo Scientific). Tryptic peptides were loaded onto a trap C18 PepMap 100 column (300 μm i.d. × 5 mm, 5 μm particle size, 100 Å pore size) and, later, eluted to an analytical column (EASY-Spray PepMap RSLC C18, 2 μm, 100 Å 75 μm × 15 cm) with a flow rate of 350 nL/min and column temperature of 40 °C. There were 500 ng of peptides loaded in buffer A (0.1% FA, 98:2% H_2_O/ACN) and separated with a buffer B (0.1% FA 98:2% ACN/H_2_O). The separation gradient started at ranges of 3–64% ACN gradient for 179 min. The voltage of electrospray was 2.0 kV, and the temperature of the capillary was 275 °C. By using full MS/DD (data dependent)–MS/MS mode, the mass spectrometry data were obtained. The AGC (Automatic Gain Control) target for the full scan MS spectra was 3 × 106 ions in the 350–1400 *m*/*z* range with an IT of 100 ms and at a resolution of 60,000 at *m*/*z* 200. Precursor ions above the 2 × 10^5^ intensity threshold were isolated with an isolation window of 1.3 *m*/*z*. The normalized collision energy was set at 28%. The MS/MS evaluations were performed at a resolution of 15,000 with an AGC target value of 10^5^ ions.

### Protein Identification and Quantification

The data were analyzed using MaxQuant version 2.0.3.0 to search using an original UniProt database of the organism *Beta vulgaris* subsp. *vulgaris* with organism id 3555. There exists only one reference proteome which is *Beta vulgaris* (UP000035740) database with total protein amount of 7679. Date of release of this database is 2013.^19^ A precursor mass tolerance of 0.5 Da, and a fragment ion mass tolerance of 5 ppm were used. The false discovery rate was set at 0.01 for peptides. Proteins were identified with at least two peptides of a minimum length of six amino acids. Proteins were quantified using the label-free quantification (LFQ) algorithm in MaxQuant. To analyze the functional protein association network of the proteomics data a web platform called STRING was used (string-db.org/).^20^

### Bioinformatics and Statistical Analysis

The label-free preprocessed proteomics data, which was obtained by MaxQuant, was analyzed and visualized by an interactive web platform, LFQ-Analyst (analyst-suite.monash-proteomics.cloud.edu.au/apps/lfq-analyst/).^21^ The adjusted *p*-value cutoff was set as 0.05. The log_2_ fold change cutoff was set as 1, and the corresponding real value of that fold change is 2. Perseus-type imputation and Benjamini Hochberg-type FDR correction were used.

## Result and Discussion

Sugar beet leaf proteins were obtained by 5 different extraction/precipitation methods. Protein extracts were first digested using trypsin and analyzed using an MS based untargeted proteomics approach. The total protein yield, relative RuBisCO content, and protein profiles were compared for each condition.

### In-Depth Sugar Beet Leaves Protein Profile

Protein extracts obtained from different methods were digested and monitored using a Q-Exactive high resolution mass spectrometer. The data were analyzed using MaxQuant using a *Beta vulgaris* background proteome. The protein IDs and gene names of all of the identified proteins and the corresponding extraction methods are provided as a table in the Supporting Information (Table S1 of the Supporting Information). Across five different processing methods, 817 proteins were identified at 1% false discovery rate (FDR). Approximately, 80% of the proteins were common at all conditions.

Hajheidari et al. previously reported approximately 500 sugar beet leaf proteins.^17^ The amount of detected protein was expected to increase after the extraction process because while the leaf itself has 20% protein in dry basis,^6^ the extracts have protein content higher than 65%.^5^ This study is the most comprehensive proteomics study to date in sugar beet leaves.

**Figure 1:**
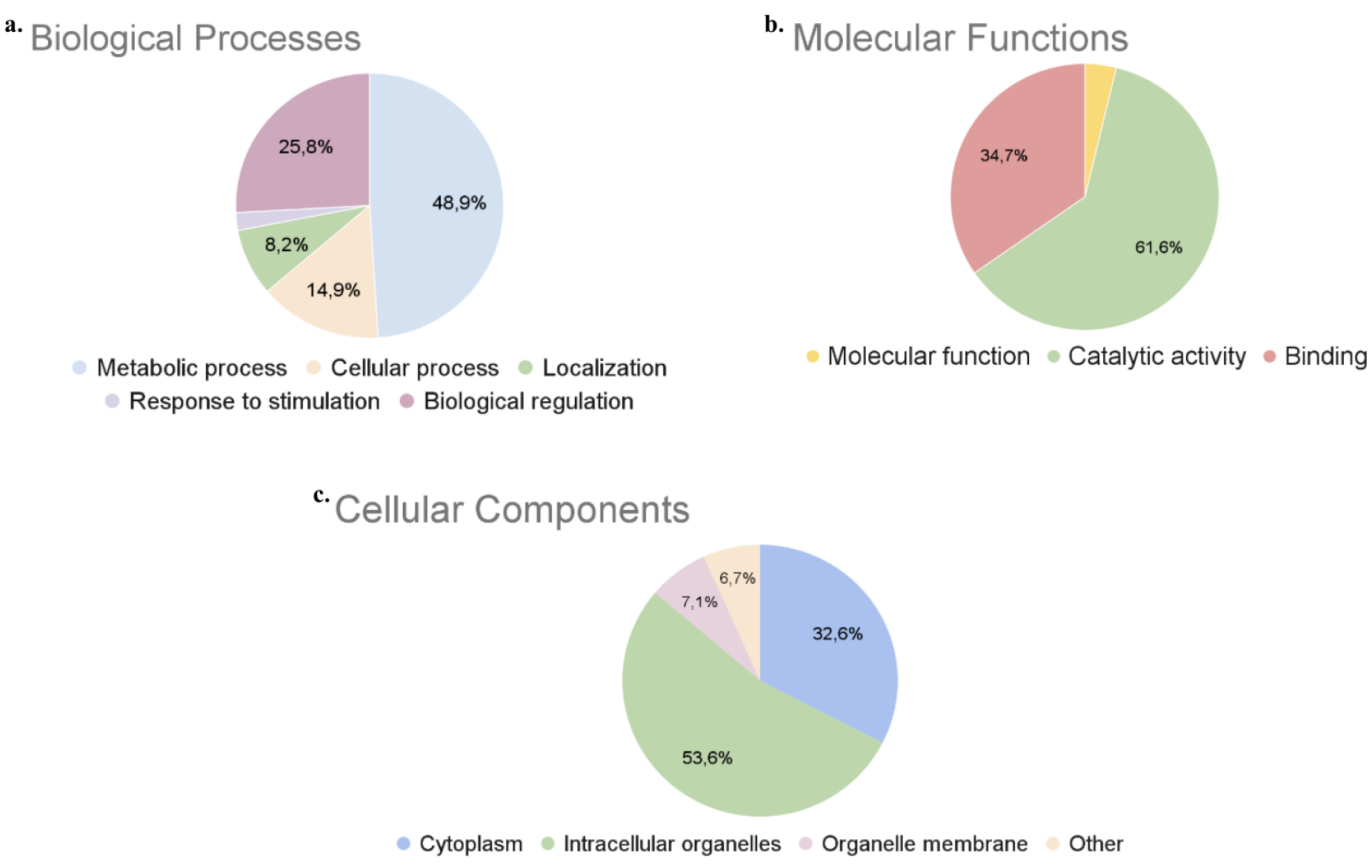
GO distribution in (a) Biological Processes, (b) Molecular Functions, and (c) Cellular Components.

To investigate the biological processes, molecular functions, and cellular components of the proteins detected in beet leaves, the protein–protein interactions were analyzed using the STRING database. The molecular functions of gene products were determined by biological processes and cellular locations of each protein.^22^ As can be seen in Figure 1, approximately half of the extracted proteins are involved in metabolic and biosynthetic biological process groups. More than 70 different proteins are included in the photosynthesis reactions that are the main part of the biological process.^13^ The other top processes are biological regulations, cell localization, and cellular processes.

The protein responsible for the most abundant enzymatic process in leaf structure is the RuBisCO protein since it yields up to 40% of total protein content in green tissue.^23^ The chloroplast is the main source of proteins in the leaf.^11^ Our results show that the majority of the proteins detected in all extraction/precipitation techniques were located in the intracellular organelles.

**Figure 2:**
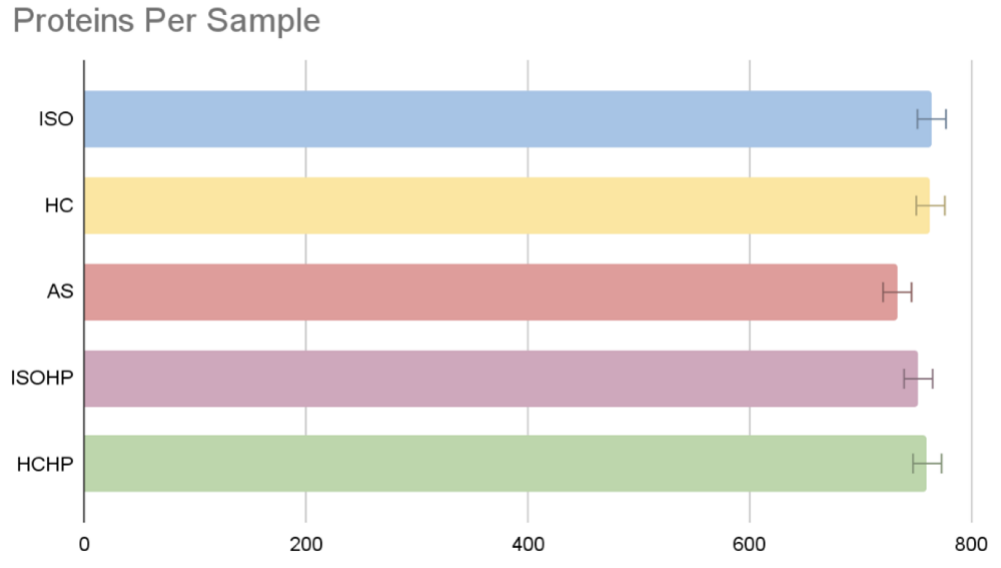
Number of extracted proteins in each extraction method.

While examining each extraction method separately, the condition of each protein group to appear in at least 3 samples out of 4 samples had been followed to find the overall protein groups for specific extraction methods, and it is seen that the number of observed protein groups varied between 730 and 770. When the individual methods are examined, the number of detected protein groups are 764, 763, 733, 752, and 760, respectively, for ISO, HC, AS, ISOHP, and HCHP (Figure 2).

After discussing the observed extracted protein amounts, the intensities and contents of the extracted proteins in each method were evaluated.

**Figure 3:**
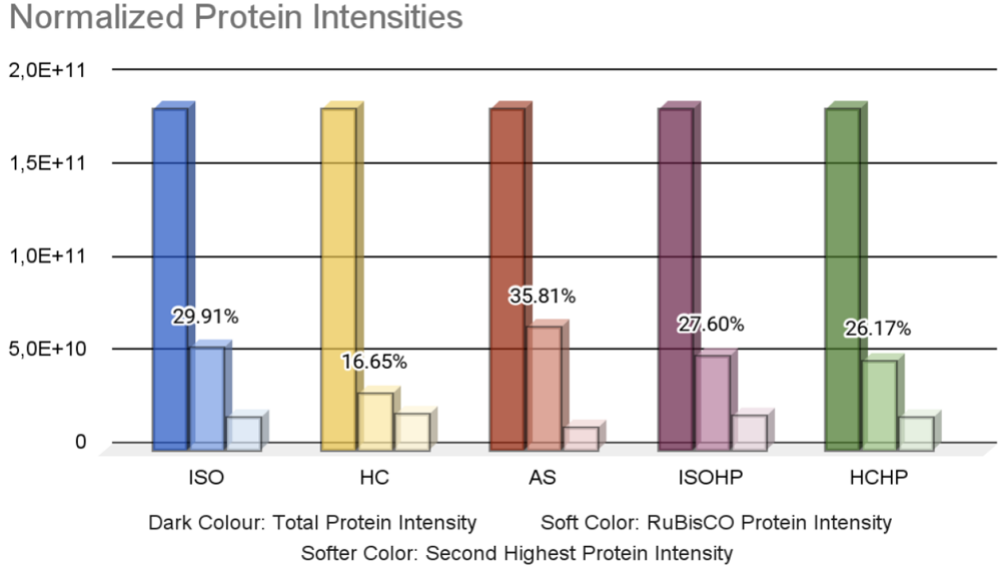
Protein intensities in each extraction method.

Normalized total protein intensities are the same in each extraction method as seen in Figure 3. However, the specific intensities of any protein group differ from method to method. RuBisCO protein is known as the most commonly found protein in leaves. Therefore, the relative intensity of extracted RuBisCO protein by each method is crucial for determining the adequacy of any extraction method that is applied. As expected the highest-intensity protein in all extraction methods is ribulose biphosphate carboxylase large chain (RuBisCO large subunit) (EC 4.1.1.39) (Protein ID: A0A023ZPS4, Gene: *rbcLcbbL*). The intensity of this protein in each extraction method differs from technique to technique slightly (Figure 3). The highest RuBisCO intensity % was observed in ammonium sulfate precipitation. Change in the ionic strength of the environment affects the solubility of the proteins. Both sulfate and ammonium ions decreased the solubility of proteins. The salting-out and hydrophobic effects result in protein aggregation and precipitation.^24^ The lowest RuBisCO intensity % was observed in the heat coagulation method. For this analysis, the total intensity value was the same. The relatively lower percentage of RuBisCO protein might be due to inefficient extraction of specific proteins from the cell matrix for that batch. Furthermore, there was no significant difference between ISO and ISOHP on total protein level. The second most abundant protein in all 5 extraction methods is ATP synthase subunit beta (EC 7.1.2.2) (Protein ID: A0A023ZRA3, Gene: *atpB*). It is an enzyme that is found in the chloroplast of sugar beet leaves and responsible for producing ATP from ADP.^25^ The fact that the two most abundant proteins were the same in all extraction techniques and the intensities of those proteins constituted a relatively large portion of the total protein intensity. Therefore it can be said that there were no significant differences between the methods in terms of intensities.

**Figure 4:**
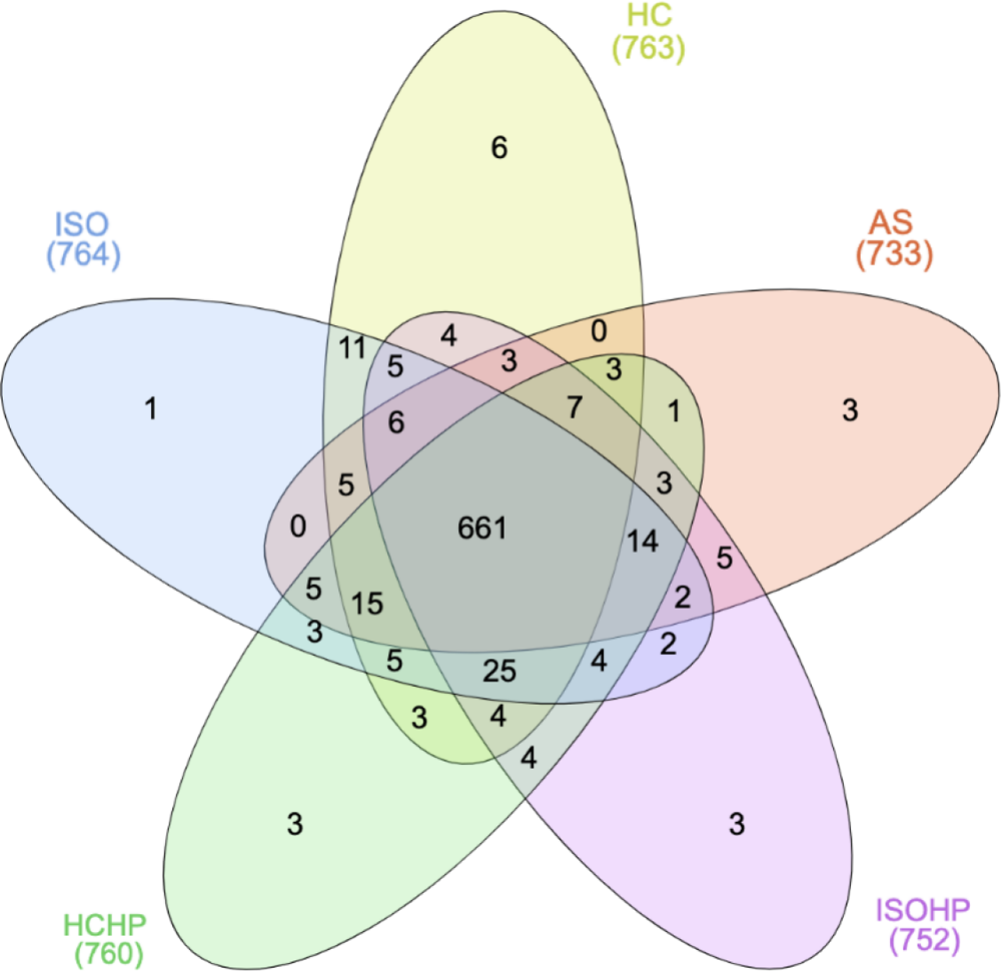
Venn diagram for overall number of extracted proteins in each extraction method (with respect to presence or absence of proteins).

In addition, a web-based tool was used to set a Venn diagram^26^ to illustrate the small difference in the number of proteins found in each extraction. 81% of these proteins (661 out of 817) were observed as common in all extraction methods (Figure 4).

The relative intensities of specific proteins extracted alone in each technique (Figure 4) were negligible since the values were lower than 0.005%.

**Figure 5:**
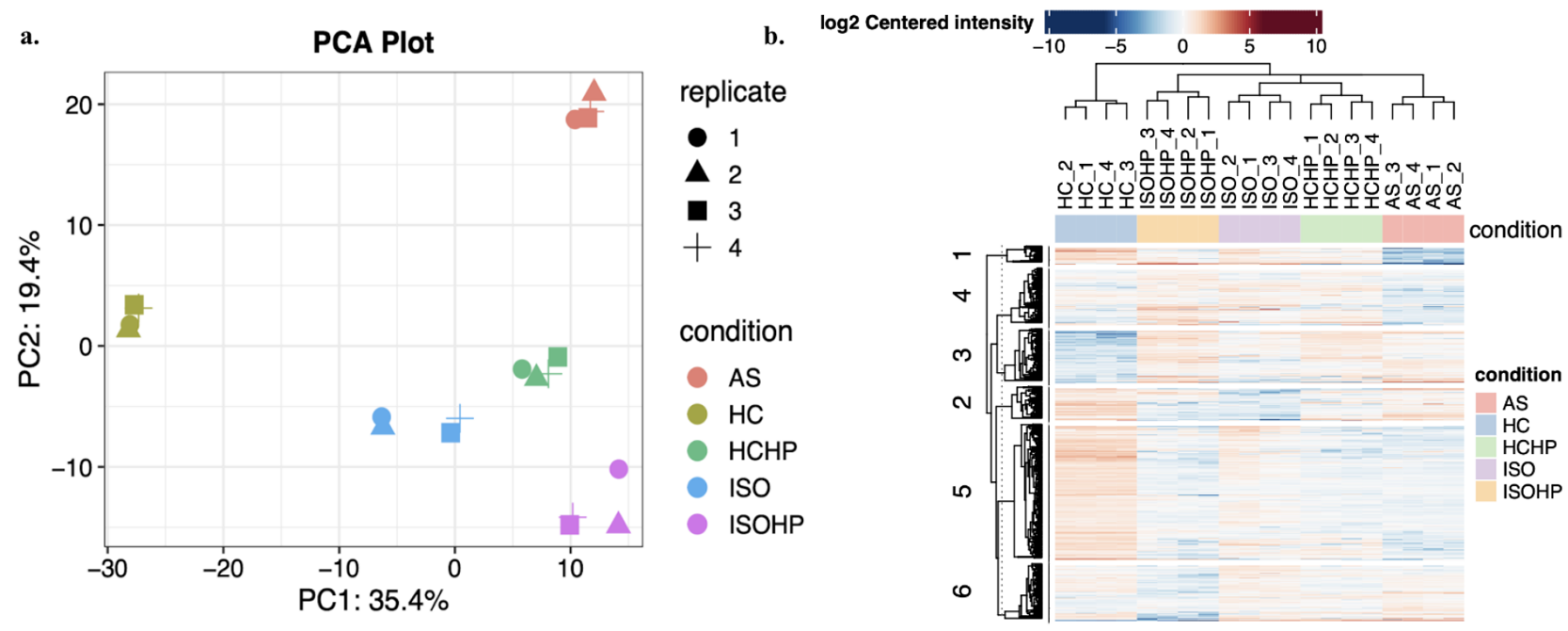
(a) PCA plot and (b) heat map of each sample in each extraction method.

Statistical analysis was performed in order to understand the differences in extraction methods. The Principle Component Analysis (PCA) and hierarchical clustering heat map of protein expression levels are shown in Figure 5. Although there is no significant difference in total protein intensities, the LFQ analysis yielded 569 proteins that differ significantly among all methods (Table S2 of the Supporting Information). The PCA plot of the components with the highest loadings is Figure 5a.^21^ The PCA of protein profiles clearly identified the different extraction methods. Therefore, low abundant proteins play an important role in the separation. The identification of low abundant proteins may be crucial from a nutritional perspective that needs to be further investigated. Since HC samples were well separated from the rest, the PCA was re-performed by excluding the HC data. The separation between the extraction methods was slightly improved (Figure S3 of the Supporting Information). Moreover, according to Figure 5b, ISOHP and HCHP have higher intensities in cluster 5 (Table S2 of the Supporting Information) than ISO and HC. The proteins located in this cluster have a function of chlorophyll binding which denotes that they are located in chloroplast, and the one of the most important target proteins which is RuBisCO is located in chloroplast. Also, while clusters 3 and 4 (Table S2 of the Supporting Information) were observed to be higher in AS, clusters 1, 2, and 6 (Table S2 of the Supporting Information) were observed to be lower. This indicated that with the AS method it is possible to get proteins located in cytoplasm and extracellular exosome with a function of proteolysis, but it is harder to obtain proteins located in several membranes which included chloroplast and mitochondrial membranes.

**Figure 6:**
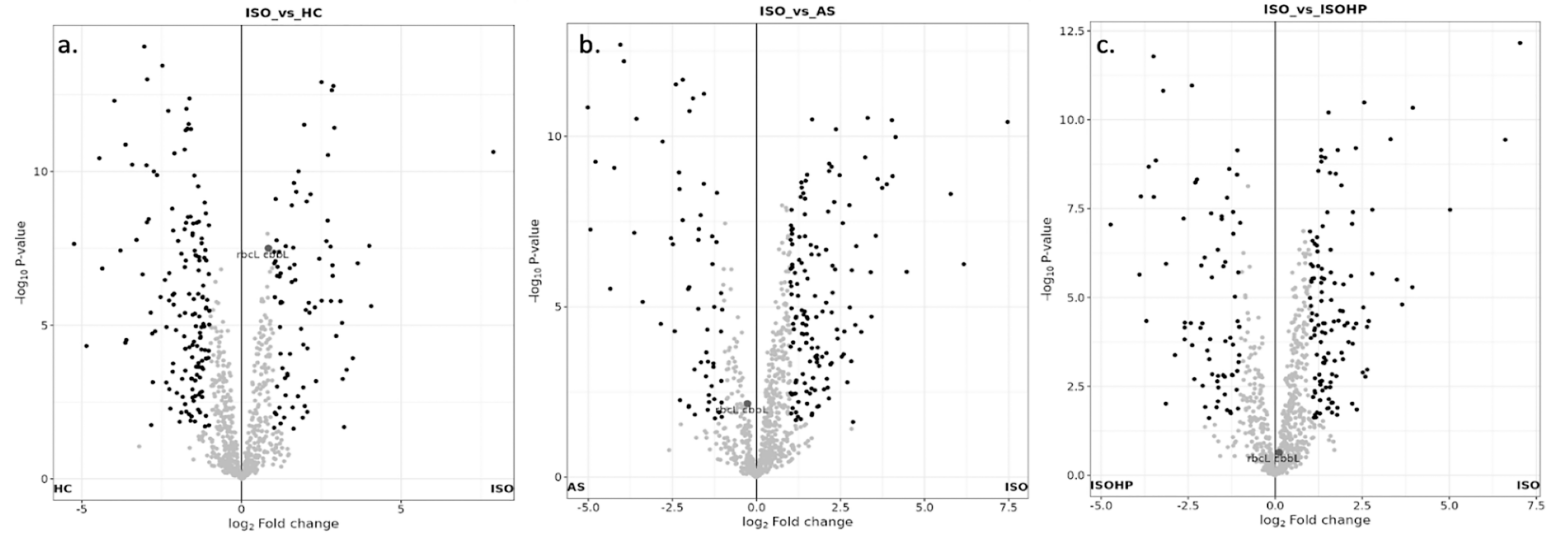
Volcano plot of (a) ISO vs HC, (b) ISO vs AS, and (c) ISO vs ISOHP.

To compare the ISO-HC and ISO-AS, LFQ-based volcano plots were examined (Figure 6). This plot shows the protein expression level changes and makes the comparison between two different groups, and the proteins were considered differentiable, showing a log_2_(fold change) of ≥1. Each point denotes one protein, and the location of the point in the axis of log_2_(fold change) indicates by which extraction technique the protein is obtained with higher intensity. If the point is in gray color, it indicates that there is no statistically significant difference between two different extraction methods, and black point indicates significant difference specific to that protein. Since the most important protein of interest is RuBisCO protein, it is marked on the plot specifically as a red dot.

In Figure 6a, the log_2_(fold change) of RuBisCO protein between ISO and HC is 1.11; since it is higher than 1 the obtained RuBisCO protein in ISO was found to be significantly higher than that in HC. It is seen from Figure 6c that there was no significant effect of high pressure on the intensity of RuBisCO proteins. This shows that, without high pressure, the methods are sufficient for extracting the RuBisCO proteins. The main goal of using high-pressure-assisted extraction was to damage the leaf tissues so that the proteins that were not accessible before would release to the solution for further precipitation, and this also explained the broader protein profile. As confirmed in Figure 6b, although there was no significant difference in terms of RuBisCO protein, a broader protein profile was obtained with ISO which was consistent with the goal of accessing more proteins through cell damage.

**Figure 7:**
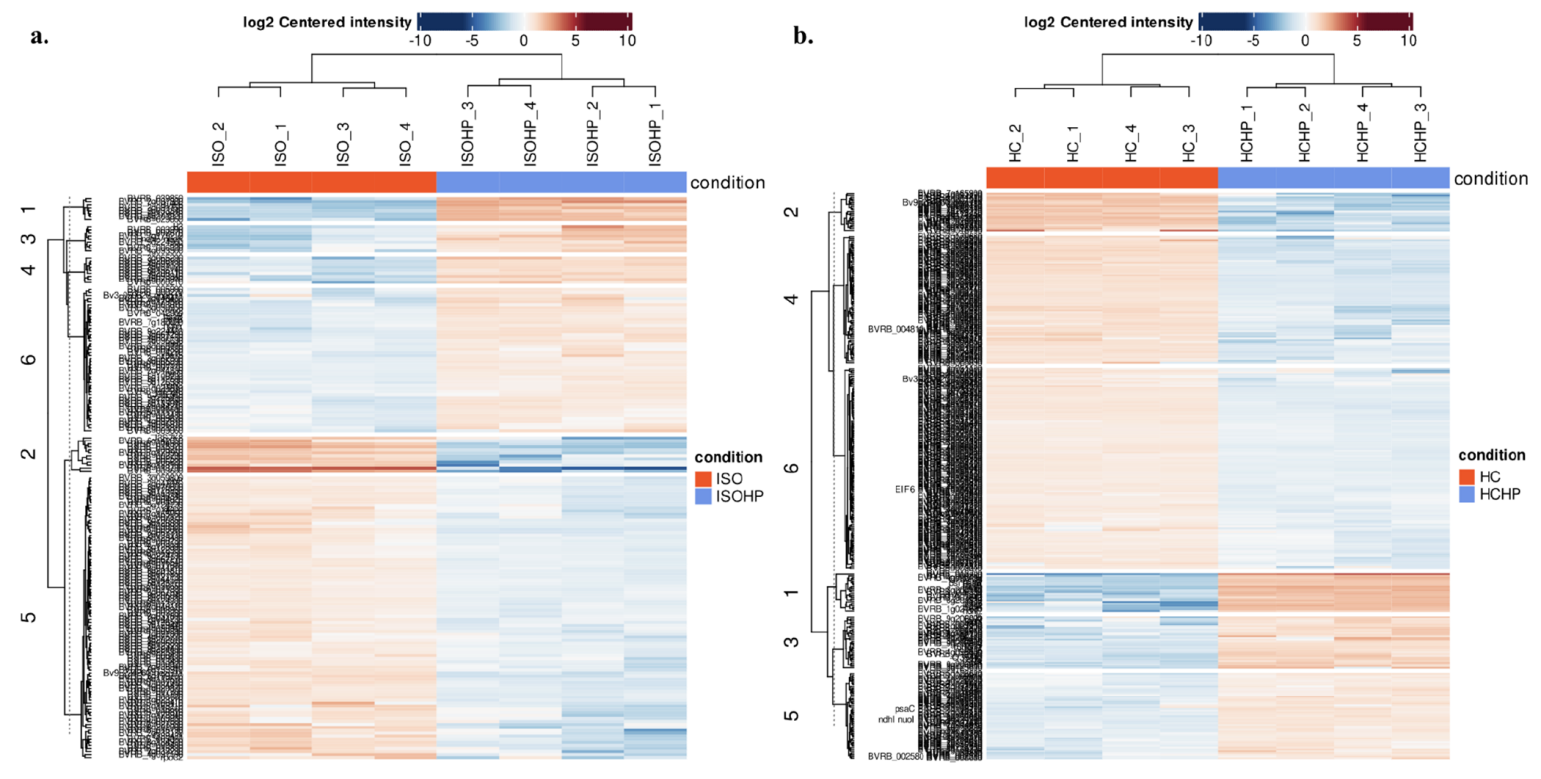
Heat map of (a) ISO vs ISOHP and (b) HC vs HCHP.

Another important aspect of this study was to investigate the effect of high pressure (HP) on protein extraction efficiency. Figure 7 shows the effect of HP on protein composition for both HC and ISO. The heat maps indicate an overview of expression of all significant (differentially expressed) proteins (rows) in both conditions (columns). In both conditions, the HP significantly increased the protein concentrations for specific cluster groups. The comparison between ISO vs ISOHP cluster 6 and comparison between HC vs HCH cluster 3 show proteins that are located in the chloroplast tissue. The specific cluster groups predominantly consisting of the proteins located in cellulose were upregulated in high-pressure-assisted extractions. As leaf proteins are embedded in the cellulosic leaf tissue, high-pressure-assisted extraction methods may damage the green fibrous tissue. Therefore, it improves the extraction efficiency.

In summary, the effect of different protein extraction/precipitation techniques on sugar beet leaves was investigated using a proteomics approach. The total protein, RuBisCO, and comprehensive protein profiles were compared using statistical approaches. With all methods combined, 817 proteins were found; 764, 763, 733, 752, and 760 proteins were obtained by isoelectric point precipitation, heat coagulation, ammonium sulfate precipitation, high-pressure-assisted isoelectric point precipitation, and high-pressure-assisted heat coagulation, respectively. The analyses yielded the most comprehensive protein coverage to date for *Beta vulgaris*. The most abundant protein was RuBisCO, and overall protein intensities were similar in all conditions. The RuBisCO content was 29.91%, 16.65%, 35.81%, 27.60%, and 26.17%, respectively, in ISO, HC, AS, ISOHP, and HCHP where HC yielded the least and AS yielded the highest amount. A total of 661 proteins out of 817 were common in each method. The differences were mainly on low abundance proteins. High-pressure-assisted methods showed elevated levels of proteins predominantly located in chloroplast due to higher extraction efficiencies. This shows that the usage of high pressure complementary to other extraction procedures can increase the amount of yielded protein. Comprehensive protein profiling is essential for the food industry as the physical and nutritional properties are highly related to protein composition. Here we showed for the first time that the extraction/precipitation methods may cause different protein profiles. The study showed that HHP was an advantageous method to increase the number of different protein groups that were extracted. However, it should be noted that HHP could also have an effect on the techno-functional properties of proteins such as emulsification, foaming, gelling, and solubility. In terms of sensorial properties, it may also result in differences compared to other methods. Economic feasibility of the process is also another aspect that needs to be addressed. These points should also be considered in further studies.

## Supporting Information Available

List of all identified proteins, list of 569 proteins that differ significantly between samples and cluster locations of each protein in heat map, and PCA analysis without HC (PDF)

## Abbreviations

ADP: adenosine diphosphate
AS: ammonium sulfate precipitation
ATP: adenosine triphosphate
GO: Gene Ontology
HC: heat coagulation
HHP: high-pressure homogenization
ISO: isoelectric point precipitation
ISOHP: high-pressure-assisted isoelectric point precipitation
ISOHC: high-pressure-assisted heat coagulation
LFQ: label free quantification
MS: mass spectrometry
PCA: principal component analysis
PEF: pulsed electric field
RuBisCO: ribulose 1,5-biphosphate carboxylase/oxygenase
US: ultrasonication

## References

[ref1] Springmann M.; Clark M.; Mason-D’Croz D.; Wiebe K.; Bodirsky B. L.; Lassaletta L.; de Vries W.; Vermeulen S. J.; Herrero M.; Carlson K. M.; et al. Options for keeping the food system within environmental limits. Nature 2018, 562, 519–525. 10.1038/s41586-018-0594-0. 30305731

[ref2] Lange W.; Brandenburg W. A.; Bock T. S. M. D. Taxonomy and cultonomy of beet (Beta vulgaris L.). Botanical Journal of the Linnean Society 1999, 130, 81–96. 10.1111/j.1095-8339.1999.tb00785.x.

[ref3] Cheesman O. D. Environmental Impacts of Sugar Production The Cultivation and Processing of Sugarcane and Sugar Beet; CABI International, 2004.

[ref4] FAO Sugarbeet | Land & Water | Food and Agriculture Organization of the United Nations. 2022,https://www.fao.org/land-water/databases-and-software/crop-information/sugarbeet/en/.

[ref5] Akyüz A.; Ersus S. Optimization of enzyme assisted extraction of protein from the sugar beet (Beta vulgaris L.) leaves for alternative plant protein concentrate production. Food Chem. 2021, 335, 335 10.1016/j.foodchem.2020.127673. 32745844

[ref6] Tamayo Tenorio A.; Schreuders F.K.G.; Zisopoulos F.K.; Boom R.M.; van der Goot A.J. Processing concepts for the use of green leaves as raw materials for the food industry. Journal of Cleaner Production 2017, 164, 736–748. 10.1016/j.jclepro.2017.06.248.

[ref7] Aramrueang N.; Zicari S. M.; Zhang R. Response Surface Optimization of Enzymatic Hydrolysis of Sugar Beet Leaves into Fermentable Sugars for Bioethanol Production. Advances in Bioscience and Biotechnology 2017, 08, 51–67. 10.4236/abb.2017.82004.

[ref8] Lammens T. M.; Franssen M. C.; Scott E. L.; Sanders J. P. Availability of protein-derived amino acids as feedstock for the production of bio-based chemicals. Biomass and Bioenergy 2012, 44, 168–181. 10.1016/j.biombioe.2012.04.021.

[ref9] Kiskini A.; Vissers A.; Vincken J. P.; Gruppen H.; Wierenga P. A. Effect of Plant Age on the Quantity and Quality of Proteins Extracted from Sugar Beet (Beta vulgaris L.) Leaves. J. Agric. Food Chem. 2016, 64, 8305–8314. 10.1021/acs.jafc.6b03095. 27750423

[ref10] Michelfelder A. J. Soy: A Complete Source of Protein. American Family Physician 2009, 79, 43–47. 19145965

[ref11] Zhang W.; Grimi N.; Jaffrin M. Y.; Ding L.; Tang B. A short review on the research progress in alfalfa leaf protein separation technology. J. Chem. Technol. Biotechnol. 2017, 92, 2894–2900. 10.1002/jctb.5364.

[ref12] Buchanan-Wollaston V. Postharvest Physiology | Senescence, Leaves. In Encyclopedia of Applied Plant Sciences, 1st ed.; Thomas B., Ed.; Academic Press, 2003; pp 808–816.

[ref13] Santamaría-Fernández M.; Lübeck M. Production of leaf protein concentrates in green biorefineries as alternative feed for monogastric animals. Animal Feed Science and Technology 2020, 268, 114605 10.1016/j.anifeedsci.2020.114605.

[ref14] Sharkey T. D. The discovery of rubisco. Journal of Experimental Botany 2023, 74, 510 10.1093/jxb/erac254. 35689795

[ref15] Kumar M.; Tomar M.; Potkule J.; Verma R.; Punia S.; Mahapatra A.; Belwal T.; Dahuja A.; Joshi S.; Berwal M. K.; Satankar V.; Bhoite A. G.; Amarowicz R.; Kaur C.; Kennedy J. F. Advances in the plant protein extraction: Mechanism and recommendations. Food Hydrocolloids 2021, 115, 106595 10.1016/j.foodhyd.2021.106595.

[ref16] Wang W.; Tai F.; Chen S. Optimizing protein extraction from plant tissues for enhanced proteomics analysis. J. Sep. Sci. 2008, 31, 2032–2039. 10.1002/jssc.200800087. 18615819

[ref17] Hajheidari M.; Abdollahian-Noghabi M.; Askari H.; Heidari M.; Sadeghian S. Y.; Ober E. S.; Hosseini Salekdeh G. Proteome analysis of sugar beet leaves under drought stress. Proteomics 2005, 5, 950–960. 10.1002/pmic.200401101. 15712235

[ref18] Webb K. M.; Broccardo C. J.; Prenni J. E.; Wintermantel W. M. Proteomic Profiling of Sugar Beet (Beta vulgaris) Leaves during Rhizomania Compatible Interactions. Proteomes 2014, 2, 208–223. 10.3390/proteomes2020208. 28250378 PMC5302737

[ref19] Dohm J. C.; Minoche A. E.; Holtgräwe D.; Capella-Gutiérrez S.; Zakrzewski F.; Tafer H.; Rupp O.; Sörensen T. R.; Stracke R.; Reinhardt R.; Goesmann A.; et al. The genome of the recently domesticated crop plant sugar beet (Beta vulgaris). Nature 2013 505:7484 2014, 505, 546–549. 10.1038/nature12817. 24352233

[ref20] Doncheva N. T.; Morris J. H.; Gorodkin J.; Jensen L. J. Cytoscape StringApp: Network Analysis and Visualization of Proteomics Data. J. Proteome Res. 2019, 18, 623–632. 10.1021/acs.jproteome.8b00702. 30450911 PMC6800166

[ref21] Shah A. D.; Goode R. J.; Huang C.; Powell D. R.; Schittenhelm R. B. Lfq-Analyst: An easy-To-use interactive web platform to analyze and visualize label-free proteomics data preprocessed with maxquant. J. Proteome Res. 2020, 19, 204–211. 10.1021/acs.jproteome.9b00496. 31657565

[ref22] Balakrishnan R.; Harris M. A.; Huntley R.; Van Auken K.; Cherry J. M. A guide to best practices for Gene Ontology (GO) manual annotation. Database: The Journal of Biological Databases and Curation 2013, 2013, bat054 10.1093/database/bat054. 23842463 PMC3706743

[ref23] Bindschedler L. V.; Cramer R. Quantitative plant proteomics. PROTEOMICS 2011, 11, 756–775. 10.1002/pmic.201000426. 21246733

[ref24] Aoki K.; Shiraki K.; Hattori T. Salt effects on the picosecond dynamics of lysozyme hydration water investigated by terahertz time-domain spectroscopy and an insight into the Hofmeister series for protein stability and solubility. Phys. Chem. Chem. Phys. 2016, 18, 15060–15069. 10.1039/C5CP06324H. 27193313

[ref25] Bateman A.; Martin M.-J.; Orchard S.; Magrane M.; Ahmad S.; Alpi E.; Bowler-Barnett E. H.; Britto R.; Bye-A-Jee H.; et al. UniProt: the Universal Protein Knowledgebase in 2023. Nucleic Acids Res. 2023, 51, D523 10.1093/nar/gkac1052. 36408920 PMC9825514

[ref26] Heberle H.; Meirelles V. G.; da Silva F. R.; Telles G. P.; Minghim R. InteractiVenn: A web-based tool for the analysis of sets through Venn diagrams. BMC Bioinformatics 2015, 16, 169 10.1186/s12859-015-0611-3. 25994840 PMC4455604

