# Supplementary material for: Proteomics Approach to Differentiate Protein Extraction Methods in Sugar Beet Leaves
Source: J Agric Food Chem. 2023 Jun 5;71(23):9157–63. doi: 10.1021/acs.jafc.2c09190 (PMC10273313; doi:10.1021/acs.jafc.2c09190)

Supporting Information

Proteomics Approach To Differentiate Protein Extraction Methods In Sugar Beet Leaves

Ece Goktayoglu,[†,¶] Mecit Halil Oztop,[†] and Sureyya Ozcan*,[‡]

†*Department of Food Engineering, Middle East Technical University, 06800, Ankara, Turkiye*

‡*Department of Chemistry, Middle East Technical University, 06800, Ankara, Turkiye*

¶*Department of Chemical Engineering, University of California Davis, 95616, Davis, California, United States*

*E-mail: sozcan@metu.edu.tr

Table S1. Table of all identified proteins

	Gene names	# Peptides	# Unique Peptides	# Amino Acids	ISO	HC	AS	ISOHP	HCHP
A0A023ZPS4	rbcL cbbL	46	43	475	FOUND	FOUND	FOUND	FOUND	FOUND
A0A023ZRA3	atpB	36	22	498	FOUND	FOUND	FOUND	FOUND	FOUND
A0A0J8FJ23	BVRB_3g055740	35	28	739	FOUND	FOUND	FOUND	FOUND	FOUND
A0A023ZQ87	atpA	24	23	507	FOUND	FOUND	FOUND	FOUND	FOUND
I6PD11		64	64	1442	FOUND	FOUND	FOUND	FOUND	FOUND
A0A0J8B5Q5	BVRB_012000	39	39	1042	FOUND	FOUND	FOUND	FOUND	FOUND
Q9AWA8	gln2 BVRB_001510	22	22	431	FOUND	FOUND	FOUND	FOUND	FOUND
O49812	BVRB_9g225010	9	8	264	FOUND	FOUND	FOUND	FOUND	FOUND
A0A0J8B8D4	BVRB_002570	10	2	267	FOUND	FOUND	FOUND	FOUND	FOUND
Q9SPH7	Prk	14	14	271	FOUND	FOUND	FOUND	FOUND	FOUND
A0A023ZR91	psbC	16	14	473	FOUND	FOUND	FOUND	FOUND	FOUND
A0A0J8B4F7	BVRB_004160	3	3	168	FOUND	FOUND	FOUND	FOUND	FOUND
A0A023ZQD7	psi psbB	17	17	508	FOUND	FOUND	FOUND	FOUND	FOUND
Q5QHN9		17	17	249	FOUND	FOUND	FOUND	FOUND	FOUND
A0A0J8CFW1	BVRB_4g091040	18	18	357	FOUND	FOUND	FOUND	FOUND	FOUND
A0A023ZPT1	petA	18	18	320	FOUND	FOUND	FOUND	FOUND	FOUND
A0A0J8BI85	BVRB_1g021580	7	7	185	FOUND	FOUND	FOUND	FOUND	FOUND
A0A0J8CTT4	BVRB_3g059720	21	21	508	FOUND	FOUND	FOUND	FOUND	FOUND
A0A023ZQ99	psbD	9	9	353	FOUND	FOUND	FOUND	FOUND	FOUND
A0A0J8CM32	BVRB_3g065500	28	28	487	FOUND	FOUND	FOUND	FOUND	FOUND
A0A0N9LU76	psaB	15	15	734	FOUND	FOUND	FOUND	FOUND	FOUND
Q7HJM1	atp1 atpA	20	19	506	FOUND	FOUND	FOUND	FOUND	FOUND
A0A0J8BUP3	BVRB_5g125910 BVRB_5g125920 BVRB_5g125930	9	9	103	FOUND	FOUND	FOUND	FOUND	FOUND
A0A0J8BDJ4	BVRB_4g096640	27	27	476	FOUND	FOUND	FOUND	FOUND	FOUND
A0A0J8BHS3	BVRB_1g022900	33	33	705	FOUND	FOUND	FOUND	FOUND	FOUND
A0A0J8B5H5	BVRB_012930	10	10	222	FOUND	FOUND	FOUND	FOUND	FOUND
A0A0J8BKM2	BVRB_9g208930	31	31	694	FOUND	FOUND	FOUND	FOUND	FOUND
A0A0J8BGW2	BVRB_2g046240 BVRB_2g046260	33	33	843	FOUND	FOUND	FOUND	FOUND	FOUND
A0A0J8BQG2	BVRB_9g206950	23	23	510	FOUND	FOUND	FOUND	FOUND	FOUND
A0A023ZQ82	psbA	10	10	353	FOUND	FOUND	FOUND	FOUND	FOUND
A0A0J8B648	BVRB_009040	11	11	302	FOUND	FOUND	FOUND	FOUND	FOUND
A0A023ZRB4	psbE	4	4	83	FOUND	FOUND	FOUND	FOUND	FOUND
A0A0J8BHE5	BVRB_2g044700	9	9	268	FOUND	FOUND	FOUND	FOUND	FOUND
A0A0J8B2V2	BVRB_008140	10	10	285	FOUND	FOUND	FOUND	FOUND	FOUND
A0A0J8B417	BVRB_005380	13	13	406	FOUND	FOUND	FOUND	FOUND	FOUND
A0A0J8E0F9	BVRB_8g201810	12	11	214	FOUND	FOUND	FOUND	FOUND	FOUND
Q9XFW8	Glu2	12	12	336	FOUND	FOUND	FOUND	FOUND	FOUND
A0A023ZQB4	atpE	12	12	134	FOUND	FOUND	FOUND	FOUND	FOUND
A0A0J8FG73	BVRB_3g065510	21	21	471	FOUND	FOUND	FOUND	FOUND	FOUND
A0A023ZR79	atpF	12	12	184	FOUND	FOUND	FOUND	FOUND	FOUND
A0A0J8B7T8	BVRB_004380	22	22	513	FOUND	FOUND	FOUND	FOUND	FOUND

Table S1. Table of all identified proteins

A0A0J8BBQ5	BVRB_6g155820	13	13	262	FOUND	FOUND	FOUND	FOUND	FOUND
A0A023ZQS5	psaA	12	12	750	FOUND	FOUND	FOUND	FOUND	FOUND
A0A0J8B7L7	BVRB_005040	8	8	266	FOUND	FOUND	FOUND	FOUND	FOUND
A0A0J8CTS7	BVRB_3g059640	9	9	181	FOUND	FOUND	FOUND	FOUND	FOUND
A0A0J8BVB1	BVRB_8g188140	11	11	342	FOUND	FOUND	FOUND	FOUND	FOUND
A0A0J8CM16	BVRB_4g087070	19	19	601	FOUND	FOUND	FOUND	FOUND	FOUND
A0A0J8BBH6	BVRB_6g156310	8	8	840	FOUND	FOUND	FOUND	FOUND	FOUND
A0A0J8B1V3	BVRB_013610	7	7	146	FOUND	FOUND	FOUND	FOUND	FOUND
A0A0J8B913	BVRB_5g126880	10	10	539	FOUND	FOUND	FOUND	FOUND	FOUND
O04054	BV-70/5	18	18	332	FOUND	FOUND	FOUND	FOUND	FOUND
A0A0J8BQL4	BVRB_9g206190	4	4	228	FOUND	FOUND	FOUND	FOUND	FOUND
A0A0J8B8V5	BVRB_5g124340	14	14	406	FOUND	FOUND	FOUND	FOUND	FOUND
A0A0J8E2K8	BVRB_6g155850	39	39	1468	FOUND	FOUND	FOUND	FOUND	FOUND
A0A0J8CL67	BVRB_4g075860	7	7	168	FOUND	FOUND	FOUND	FOUND	FOUND
A0A023ZQW4	petB	6	6	231	FOUND	FOUND	FOUND	FOUND	FOUND
A0A0J8B9V6	BVRB_5g124140	9	9	241	FOUND	FOUND	FOUND	FOUND	FOUND
A0A0J8BD59	BVRB_5g123260	7	7	170	FOUND	FOUND	FOUND	FOUND	FOUND
A0A0J8B5C7	BVRB_001060	4	4	208	FOUND	FOUND	FOUND	FOUND	FOUND
A0A0J8CTT9	BVRB_3g059770	16	16	413	FOUND	FOUND	FOUND	FOUND	FOUND
A0A0J8B6M2	BVRB_007160	12	12	235	FOUND	FOUND	FOUND	FOUND	FOUND
A0A0J8DSR1	BVRB_027930	5	3	237	FOUND	FOUND	FOUND	FOUND	FOUND
A0A0J8B7Z9	BVRB_002330	26	26	572	FOUND	FOUND	FOUND	FOUND	FOUND
A0A0J8B9R8	BVRB_4g096280	21	21	452	FOUND	FOUND	FOUND	FOUND	FOUND
A0A0J8CV90	BVRB_3g055710	13	13	147	FOUND	FOUND	FOUND	FOUND	FOUND
A0A0J8BAX7	BVRB_4g096300	17	17	388	FOUND	FOUND	FOUND	FOUND	FOUND
A0A0J8CQA6	BVRB_4g075650	3	3	150	FOUND	FOUND	FOUND	FOUND	FOUND
A0A0J8B744	BVRB_7g179200	9	9	285	FOUND	FOUND	FOUND	FOUND	FOUND
A0A0J8CW97	BVRB_2g033360	1	1	511	FOUND	FOUND	FOUND	FOUND	FOUND
A0A0J8CS02	BVRB_3g065490	3	3	163	FOUND	FOUND	FOUND	FOUND	FOUND
A0A0J8B7I9	BVRB_7g177710	4	4	216	FOUND	FOUND	FOUND	FOUND	FOUND
A0A0J8BDD0	BVRB_4g096860	4	4	186	FOUND	FOUND	FOUND	FOUND	FOUND
A0A0J8B525	BVRB_000430	3	3	128	FOUND	FOUND	FOUND	FOUND	FOUND
A0A0J8BKN7	BVRB_9g208890	9	2	279	FOUND	FOUND	FOUND	FOUND	FOUND
A0A0J8B741	BVRB_006710	9	9	438	FOUND	FOUND	FOUND	FOUND	FOUND
A0A023ZPX6	psaC BVRB_7g168100	1	1	81	FOUND	FOUND	FOUND	FOUND	FOUND
A0A0J8B972	BVRB_9g225490	14	14	168	FOUND	FOUND	FOUND	FOUND	FOUND
A0A0J8B6R5	BVRB_007930	15	15	1120	FOUND	FOUND	FOUND	FOUND	FOUND
A0A0J8BC48	BVRB_3g067500	3	3	106	FOUND	FOUND	FOUND	FOUND	FOUND
A0A0J8DXV5	BVRB_005770	5	5	579	FOUND	FOUND	FOUND	FOUND	FOUND
Q9XFW7	Ch4	7	7	265	FOUND	FOUND	FOUND	FOUND	FOUND
A0A0J8BM33	BVRB_9g206220	11	11	196	FOUND	FOUND	FOUND	FOUND	FOUND
A0A0J8DZ23	BVRB_001880	5	5	184	FOUND	FOUND	FOUND	FOUND	FOUND
A0A0J8CS67	BVRB_3g050700	9	9	211	FOUND	FOUND	FOUND	FOUND	FOUND
A0A0J8B411	BVRB_005520	6	6	152	FOUND	FOUND	FOUND	FOUND	FOUND
A0A0J8B8R9	BVRB_001360	4	4	358	FOUND	FOUND	FOUND	FOUND	FOUND

Table S1. Table of all identified proteins

A0A0J8BH59	BVRB_039850	1	1	192	FOUND	FOUND	NOT FOUND	FOUND	FOUND
A0A023ZQG2	rps7	7	7	155	FOUND	FOUND	FOUND	FOUND	FOUND
A0A0J8BBV5	BVRB_5g127120	16	16	396	FOUND	FOUND	FOUND	FOUND	FOUND
A0A0J8B797	BVRB_006360	4	1	138	FOUND	FOUND	FOUND	FOUND	FOUND
A0A0J8E7U9	BVRB_2g047050	25	25	748	FOUND	FOUND	FOUND	FOUND	FOUND
A0A0J8DXX0	BVRB_005630	6	6	230	FOUND	FOUND	FOUND	FOUND	FOUND
A0A023ZQE9	rps8	4	4	134	FOUND	FOUND	FOUND	FOUND	FOUND
A0A0J8E4J4	BVRB_4g096340	7	7	218	FOUND	FOUND	FOUND	FOUND	FOUND
A0A0J8B8Y7	BVRB_5g123970	7	7	199	FOUND	FOUND	FOUND	FOUND	FOUND
A0A0J8B5T5	BVRB_8g201590	7	7	192	FOUND	FOUND	FOUND	FOUND	FOUND
A0A0J8B7M5	BVRB_004870	10	10	262	FOUND	FOUND	FOUND	FOUND	FOUND
A0A0J8B8J2	BVRB_000070	8	8	300	FOUND	FOUND	FOUND	FOUND	FOUND
A0A0J8BKL1	BVRB_9g209080	14	14	423	FOUND	FOUND	FOUND	FOUND	FOUND
A0A0J8B6A3	BVRB_009290	11	11	513	FOUND	FOUND	FOUND	FOUND	FOUND
C7DYC4		5	5	286	FOUND	FOUND	FOUND	FOUND	FOUND
A0A0J8BCR7	BVRB_5g122980	11	11	494	FOUND	FOUND	FOUND	FOUND	FOUND
Q9FEH7	H(+)-ATPase	8	8	186	FOUND	FOUND	FOUND	FOUND	FOUND
A0A0J8B475	BVRB_004950	10	10	246	FOUND	FOUND	FOUND	FOUND	FOUND
A0A0J8B5S9	BVRB_9g225860	10	10	396	FOUND	FOUND	FOUND	FOUND	FOUND
A0A0J8B6U7	BVRB_006030	19	19	689	FOUND	FOUND	FOUND	FOUND	FOUND
A0A0J8BDD7	BVRB_2g044840	12	12	244	FOUND	FOUND	FOUND	FOUND	FOUND
A0A0J8B8N6	BVRB_000920	6	6	267	FOUND	FOUND	FOUND	FOUND	FOUND
A0A023ZQU4	psbL	1	1	38	FOUND	FOUND	FOUND	FOUND	FOUND
A0A0J8FKL0	BVRB_3g050740	10	10	193	FOUND	FOUND	FOUND	FOUND	FOUND
A0A0J8E6N8	BVRB_3g067650	12	12	237	FOUND	FOUND	FOUND	FOUND	FOUND
A0A023ZRD6	rpl14	5	5	121	FOUND	FOUND	FOUND	FOUND	FOUND
A0A0J8CWJ3	BVRB_3g050890	6	6	202	FOUND	FOUND	FOUND	FOUND	FOUND
A0A0J8CQK4	BVRB_3g055550	7	7	236	FOUND	FOUND	FOUND	FOUND	FOUND
A0A0J8E1W4	BVRB_7g178720	13	13	332	FOUND	FOUND	FOUND	FOUND	FOUND
A0A023ZR97	rps4	12	12	201	FOUND	FOUND	FOUND	FOUND	FOUND
A0A0J8B5H2	BVRB_012980	11	11	617	FOUND	FOUND	FOUND	FOUND	FOUND
A0A0J8E1T9	BVRB_7g179210	2	2	254	FOUND	FOUND	FOUND	FOUND	FOUND
A0A0J8CWG9	BVRB_2g033010	4	4	270	FOUND	FOUND	FOUND	FOUND	FOUND
A0A0J8BDE4	BVRB_3g068040	14	14	397	FOUND	FOUND	FOUND	FOUND	FOUND
A0A0J8E469	BVRB_4g097610	15	15	325	FOUND	FOUND	FOUND	FOUND	FOUND
A0A0J8C3H6	BVRB_6g144110	17	17	612	FOUND	FOUND	FOUND	FOUND	FOUND
A0A0J8B5P2	BVRB_010930	12	12	437	FOUND	FOUND	FOUND	FOUND	FOUND
A0A0J8E1T2	BVRB_7g179160	8	8	178	FOUND	FOUND	FOUND	FOUND	FOUND
A0A0J8BW28	BVRB_8g185040	5	5	158	FOUND	FOUND	FOUND	FOUND	FOUND
A0A0J8B9R6	BVRB_8g201670	9	9	2129	FOUND	FOUND	FOUND	FOUND	FOUND
A0A0J8B3V6	BVRB_004290	9	9	276	FOUND	FOUND	FOUND	FOUND	FOUND
A0A0J8B2L2	BVRB_010820	11	11	239	FOUND	FOUND	FOUND	FOUND	FOUND
V5QQV5	Bv3_050620_qhag.t1	7	7	152	FOUND	FOUND	FOUND	FOUND	FOUND
A0A0J8B8Z8	BVRB_9g225070	20	20	787	FOUND	FOUND	FOUND	FOUND	FOUND
A0A0J8B3D2	BVRB_006450	10	10	408	FOUND	FOUND	FOUND	FOUND	FOUND

Table S1. Table of all identified proteins

A0A0J8FB46	BVRB_4g087150	1	1	35	FOUND	FOUND	FOUND	FOUND	FOUND
A0A0J8B555	BVRB_013700	1	1	71	FOUND	NOT FOUND	FOUND	NOT FOUND	FOUND
A0A0J8B272	BVRB_011660	1	1	165	FOUND	FOUND	FOUND	FOUND	FOUND
A0A0J8BPT8	BVRB_9g209070	10	10	220	FOUND	FOUND	FOUND	FOUND	FOUND
A0A023ZPV8	rps3	9	9	218	FOUND	FOUND	FOUND	FOUND	FOUND
A0A0J8B2D3	BVRB_009840	7	7	477	FOUND	FOUND	FOUND	FOUND	FOUND
A0A0J8BQJ1	BVRB_9g206300	20	20	597	FOUND	FOUND	FOUND	FOUND	FOUND
A0A0J8B447	BVRB_003210	18	18	675	FOUND	FOUND	FOUND	FOUND	FOUND
A0A0J8CG82	BVRB_5g111270	18	18	457	FOUND	FOUND	FOUND	FOUND	FOUND
A0A0J8BH02	BVRB_2g046170	10	10	495	FOUND	FOUND	FOUND	FOUND	FOUND
A0A0J8B2V7	BVRB_008060	9	9	483	FOUND	FOUND	FOUND	FOUND	FOUND
A0A0J8B611	BVRB_9g224950	9	9	245	FOUND	FOUND	FOUND	FOUND	FOUND
A0A0J8BGM4	BVRB_2g047460	12	12	639	FOUND	FOUND	FOUND	FOUND	FOUND
A0A0J8B3S8	BVRB_004790	9	9	237	FOUND	FOUND	FOUND	FOUND	FOUND
A0A0N9LT71	petD	1	1	158	FOUND	FOUND	FOUND	FOUND	FOUND
A0A0J8CGH0	BVRB_4g090920	4	4	667	FOUND	FOUND	FOUND	FOUND	FOUND
A0A0J8B665	BVRB_9g224840	6	6	641	FOUND	FOUND	FOUND	FOUND	FOUND
A0A0J8B936	BVRB_5g126750	10	10	197	FOUND	FOUND	FOUND	FOUND	FOUND
A0A0J8BE74	BVRB_2g047020	6	6	168	FOUND	FOUND	FOUND	FOUND	FOUND
A0A0J8BVF1	BVRB_8g187760	11	11	394	FOUND	FOUND	FOUND	FOUND	FOUND
A0A0J8ER86	BVRB_7g168150	7	7	467	FOUND	FOUND	FOUND	FOUND	FOUND
A0A0J8B2F5	BVRB_011630	10	10	309	FOUND	FOUND	FOUND	FOUND	FOUND
A0A0J8B483	BVRB_003100	10	10	472	FOUND	FOUND	FOUND	FOUND	FOUND
A0A023ZPY0	ndhH nuoD	18	18	393	FOUND	FOUND	FOUND	FOUND	FOUND
A0A0J8BZW3	BVRB_7g168220	12	12	547	FOUND	FOUND	FOUND	FOUND	FOUND
A0A0J8FG58	BVRB_3g065360	12	12	495	FOUND	FOUND	FOUND	FOUND	FOUND
A0A0J8B6X8	BVRB_007450	10	10	366	FOUND	FOUND	FOUND	FOUND	FOUND
A0A0J8CS99	BVRB_3g050950	9	9	365	FOUND	FOUND	FOUND	FOUND	FOUND
A0A0J8CS77	BVRB_3g050790	24	24	913	FOUND	FOUND	FOUND	FOUND	FOUND
A0A0J7YPI0	BVRB_035900	3	1	220	FOUND	FOUND	FOUND	FOUND	FOUND
A0A0J8BB86	BVRB_4g095330	8	8	379	FOUND	FOUND	FOUND	FOUND	FOUND
A0A0J8CVC3	BVRB_3g056080	13	13	645	FOUND	FOUND	FOUND	FOUND	FOUND
A0A0J8ER97	BVRB_7g168330	12	12	260	FOUND	FOUND	FOUND	FOUND	FOUND
A0A0J8B5V0	BVRB_8g201390	3	3	259	FOUND	FOUND	FOUND	FOUND	FOUND
A0A0J7YMB8	BVRB_042900	2	1	136	FOUND	FOUND	FOUND	FOUND	FOUND
A0A023ZQC6	psbF	1	1	39	FOUND	FOUND	FOUND	FOUND	FOUND
A0A0J8CM87	BVRB_3g065940	17	17	1001	FOUND	FOUND	FOUND	FOUND	FOUND
A0A0J8E851	BVRB_2g046040	12	12	212	FOUND	FOUND	FOUND	FOUND	FOUND
A0A0J8DZD4	BVRB_000910	8	8	333	FOUND	FOUND	FOUND	FOUND	FOUND
A0A0J8BF21	BVRB_1g023250	4	4	214	FOUND	FOUND	FOUND	FOUND	FOUND
A0A0J8B4P4	BVRB_014940	13	13	396	FOUND	FOUND	FOUND	FOUND	FOUND
A0A0J8E2V8	BVRB_5g127150	7	7	379	FOUND	FOUND	FOUND	FOUND	FOUND
A0A0J8BKL6	BVRB_9g209130	5	5	182	FOUND	FOUND	FOUND	FOUND	FOUND
A0A0J8CVA6	BVRB_3g055900	6	6	210	FOUND	FOUND	FOUND	FOUND	FOUND
A0A0J8E823	BVRB_2g046340	21	21	965	FOUND	FOUND	FOUND	FOUND	FOUND

Table S1. Table of all identified proteins

A0A0J8CFT3	BVRB_5g113700	14	14	692	FOUND	FOUND	FOUND	FOUND	FOUND
A0A0J8EYC2	BVRB_6g142650	2	2	353	FOUND	FOUND	FOUND	FOUND	FOUND
A0A0J8B4V3	BVRB_003120	12	12	294	FOUND	FOUND	FOUND	FOUND	FOUND
A0A0J8FEH2	BVRB_4g075850	15	15	421	FOUND	FOUND	FOUND	FOUND	FOUND
A0A0J8CL57	BVRB_4g075790	7	7	313	FOUND	FOUND	FOUND	FOUND	FOUND
A0A0J8B2U8	BVRB_008230	9	9	539	FOUND	FOUND	FOUND	FOUND	FOUND
A0A0J8B8N3	BVRB_5g125170	7	7	216	FOUND	FOUND	FOUND	FOUND	FOUND
A0A0J8B508	BVRB_000220	6	6	208	FOUND	FOUND	FOUND	FOUND	FOUND
A0A0J8BCW0	BVRB_2g047170	7	7	217	FOUND	FOUND	FOUND	FOUND	FOUND
A0A0J8B6S1	BVRB_7g180020	7	7	252	FOUND	FOUND	FOUND	FOUND	FOUND
A0A0J8E9M3	BVRB_1g020870	4	4	760	FOUND	FOUND	FOUND	FOUND	FOUND
A0A0J8B3I8	BVRB_007250	12	12	469	FOUND	FOUND	FOUND	FOUND	FOUND
A0A0J8BEG8	BVRB_4g093140	6	6	264	FOUND	FOUND	FOUND	FOUND	FOUND
A0A0J8E360	BVRB_5g126250	13	13	474	FOUND	FOUND	FOUND	FOUND	FOUND
A0A0J8E055	BVRB_9g224730	12	12	321	FOUND	FOUND	FOUND	FOUND	FOUND
A0A0J8B6D7	BVRB_009080	10	10	245	FOUND	FOUND	FOUND	FOUND	FOUND
A0A0J8B9Z0	BVRB_8g200740	10	10	375	FOUND	FOUND	FOUND	FOUND	FOUND
A0A0J8C427	BVRB_6g141790	7	7	270	FOUND	FOUND	FOUND	FOUND	FOUND
A0A0J8BD29	BVRB_5g122970	9	7	442	FOUND	FOUND	FOUND	FOUND	FOUND
A0A023ZPP8	rps16	4	4	88	FOUND	FOUND	FOUND	FOUND	FOUND
A0A0J8CM43	BVRB_3g065590	5	5	253	FOUND	FOUND	FOUND	FOUND	FOUND
A0A0J8BC28	BVRB_5g125670	11	11	318	FOUND	FOUND	FOUND	FOUND	FOUND
A0A0J8BAH7	BVRB_4g097450	7	7	142	FOUND	FOUND	FOUND	FOUND	FOUND
A0A0J8BDJ6	BVRB_2g045440	8	8	328	FOUND	FOUND	FOUND	FOUND	FOUND
A0A0J8E1V4	BVRB_7g178570	10	10	329	FOUND	FOUND	FOUND	FOUND	FOUND
A0A0J8EMK8	BVRB_8g185200	9	9	419	FOUND	FOUND	FOUND	FOUND	FOUND
A0A0J8BDH5	BVRB_4g095590	10	10	461	FOUND	FOUND	FOUND	FOUND	FOUND
Q9MF87	atp6	1	1	257	FOUND	FOUND	NOT FOUND	FOUND	FOUND
A0A0J8E325	BVRB_5g126610	7	7	256	FOUND	FOUND	FOUND	FOUND	FOUND
A0A0J8BB71	BVRB_3g070220	15	15	617	FOUND	FOUND	FOUND	FOUND	FOUND
A0A0J8BAD9	BVRB_7g180450	10	10	291	FOUND	FOUND	FOUND	FOUND	FOUND
A0A023ZQV6	clpP	1	1	195	FOUND	FOUND	FOUND	FOUND	FOUND
A0A0J8BC72	BVRB_5g125980	8	8	324	FOUND	FOUND	FOUND	FOUND	FOUND
A0A0J8B7W0	BVRB_004200	3	3	106	FOUND	FOUND	FOUND	FOUND	FOUND
A0A0J8BDB7	BVRB_2g045680	9	9	533	FOUND	FOUND	FOUND	FOUND	FOUND
Q9AXD1	gln1	9	9	356	FOUND	FOUND	FOUND	FOUND	FOUND
A0A0J8BHE8	BVRB_2g045530	12	12	547	FOUND	FOUND	FOUND	FOUND	FOUND
A0A1U9XPM6		15	15	716	FOUND	FOUND	FOUND	FOUND	FOUND
A0A0J8CI37	BVRB_5g105640	16	5	421	FOUND	FOUND	FOUND	FOUND	FOUND
Q96544	cit1	10	10	437	FOUND	FOUND	FOUND	FOUND	FOUND
A0A0J8B4E8	BVRB_002490	4	2	123	FOUND	FOUND	FOUND	FOUND	FOUND
A0A0J8BEJ3	BVRB_4g093360	14	14	796	FOUND	FOUND	FOUND	FOUND	FOUND
A0A0J8B661	BVRB_008960	4	4	557	FOUND	FOUND	FOUND	FOUND	FOUND
A0A0J8BB80	BVRB_7g177690	16	16	949	FOUND	FOUND	FOUND	FOUND	FOUND
A0A0J8B3L8	BVRB_005460	13	13	487	FOUND	FOUND	FOUND	FOUND	FOUND

Table S1. Table of all identified proteins

A0A0J8B708	BVRB_007030	15	15	715	FOUND	FOUND	FOUND	FOUND	FOUND
A0A0J8BQR3	BVRB_9g206010	8	4	213	FOUND	FOUND	FOUND	FOUND	FOUND
A0A0J8B4F5	BVRB_002580 BVRB_002590	9	1	268	FOUND	FOUND	FOUND	FOUND	FOUND
A0A023ZR06	rpl2	7	7	274	FOUND	FOUND	FOUND	FOUND	FOUND
A0A0J8B5L7	BVRB_012430	3	3	121	FOUND	FOUND	FOUND	FOUND	FOUND
A0A0J8BE08	BVRB_1g022600	14	14	604	FOUND	FOUND	FOUND	FOUND	FOUND
A0A0J8BGV2	BVRB_2g046160	16	16	538	FOUND	FOUND	FOUND	FOUND	FOUND
A0A023ZQA3	ndhE nuoK	1	1	101	FOUND	FOUND	FOUND	FOUND	FOUND
A0A0J8CWT5	BVRB_3g050510	9	9	265	FOUND	FOUND	FOUND	FOUND	FOUND
A0A023ZQX5	rps19	6	6	92	FOUND	FOUND	FOUND	FOUND	FOUND
Q9MFF5	nad9	10	10	192	FOUND	FOUND	FOUND	FOUND	FOUND
A0A0J8BD81	BVRB_2g046080	11	11	1209	FOUND	FOUND	FOUND	FOUND	FOUND
A0A0J8E3G3	BVRB_5g125100	8	6	453	FOUND	FOUND	FOUND	FOUND	FOUND
A0A0J8DX57	BVRB_008150	10	10	432	FOUND	FOUND	FOUND	FOUND	FOUND
A0A0J8EMH9	BVRB_8g184860	5	5	114	FOUND	FOUND	FOUND	FOUND	FOUND
A0A0J8BEA4	BVRB_4g093950	12	12	434	FOUND	FOUND	FOUND	FOUND	FOUND
A0A0J8B5M2	BVRB_011430	2	2	137	FOUND	FOUND	FOUND	FOUND	FOUND
A0A0J8BGJ7	BVRB_2g047150	9	9	351	FOUND	FOUND	FOUND	FOUND	FOUND
A0A0J8BGE0	BVRB_2g047840	10	10	451	FOUND	FOUND	FOUND	FOUND	FOUND
A0A023ZQH2	ndhI nuoI BVRB_5g124780	5	5	170	FOUND	FOUND	FOUND	FOUND	FOUND
A0A0J8B6L3	BVRB_8g201270	10	10	373	FOUND	FOUND	FOUND	FOUND	FOUND
A0A0J8B9E3	BVRB_5g125850	9	9	267	FOUND	FOUND	FOUND	FOUND	FOUND
A0A0J8B5V5	BVRB_009820	13	13	469	FOUND	FOUND	FOUND	FOUND	FOUND
A0A0J8BHR2	BVRB_1g022760	9	9	331	FOUND	FOUND	FOUND	FOUND	FOUND
A0A0J8CBR1	BVRB_5g110140	1	1	125	FOUND	FOUND	FOUND	FOUND	FOUND
A0A0J8B6I6	BVRB_007700	3	3	262	FOUND	FOUND	FOUND	FOUND	FOUND
Q9MF86	nad7	11	11	391	FOUND	FOUND	FOUND	FOUND	FOUND
A0A0J8CBN4	BVRB_5g110230	5	1	340	FOUND	FOUND	FOUND	FOUND	FOUND
A0A0J8CG74	BVRB_5g111190	3	3	93	FOUND	FOUND	FOUND	FOUND	FOUND
A0A0J8DZ93	BVRB_001050	12	12	419	FOUND	FOUND	FOUND	FOUND	FOUND
A0A0J8B4D2	BVRB_016180	3	3	164	FOUND	FOUND	FOUND	FOUND	FOUND
A0A0J8B5Z4	BVRB_009480	4	4	188	FOUND	FOUND	FOUND	FOUND	FOUND
A0A0J8BFV0	BVRB_3g067850	7	7	198	FOUND	FOUND	FOUND	FOUND	FOUND
A0A0N9LTA0	rpl16	4	4	136	FOUND	FOUND	FOUND	FOUND	FOUND
A0A0J8DW58	BVRB_012800	6	6	286	FOUND	FOUND	FOUND	FOUND	FOUND
A0A0J8CWK9	BVRB_3g051020	3	3	293	FOUND	FOUND	FOUND	FOUND	FOUND
A0A0J8E3H4	BVRB_5g124950	1	1	189	FOUND	FOUND	FOUND	FOUND	FOUND
A0A0J8BD51	BVRB_2g046380	5	5	146	FOUND	FOUND	FOUND	FOUND	FOUND
A0A0J8E4R6	BVRB_4g095180	7	7	292	FOUND	FOUND	FOUND	FOUND	FOUND
A0A0J8BEJ7	BVRB_4g093390	7	7	268	FOUND	FOUND	FOUND	FOUND	FOUND
A0A0J8CLA4	BVRB_4g076280	16	16	976	FOUND	FOUND	FOUND	FOUND	FOUND
A0A0J8B6G4	BVRB_8g201820	4	4	132	FOUND	FOUND	FOUND	FOUND	FOUND
A0A0J8B2X7	BVRB_009090	13	13	911	FOUND	FOUND	FOUND	FOUND	FOUND
A0A7G2RMM8	BVRB_8g200370	1	1	367	FOUND	FOUND	FOUND	FOUND	FOUND
A0A0J8CIE0	BVRB_4g083560	12	12	558	FOUND	FOUND	FOUND	FOUND	FOUND

Table S1. Table of all identified proteins

A0A0J8E6V5	BVRB_3g067310	5	5	316	FOUND	FOUND	FOUND	FOUND	FOUND
A0A0J8B8U9	BVRB_000690	7	7	362	FOUND	FOUND	FOUND	FOUND	FOUND
A0A0J8CWM8	BVRB_2g031500	8	8	255	FOUND	FOUND	FOUND	FOUND	FOUND
A0A0J8E4V4	BVRB_4g095070	9	9	529	FOUND	FOUND	FOUND	FOUND	FOUND
A0A0J8BM38	BVRB_9g205950	8	8	317	FOUND	FOUND	FOUND	FOUND	FOUND
A0A0N9LMG9	clpP	2	2	195	FOUND	FOUND	FOUND	FOUND	FOUND
A0A0J8C157	EIF6 BVRB_7g164990	4	4	245	FOUND	FOUND	NOT FOUND	FOUND	FOUND
A0A0J8B848	BVRB_5g126960	17	2	1072	FOUND	FOUND	FOUND	FOUND	FOUND
A0A0J8B5W3	BVRB_011370	9	9	653	FOUND	FOUND	FOUND	FOUND	FOUND
A0A0J8B6M7	BVRB_7g180360	6	6	334	FOUND	FOUND	FOUND	FOUND	FOUND
A0A0J8DYK7	BVRB_003260	6	6	323	FOUND	FOUND	FOUND	FOUND	FOUND
A0A0J8B8E4	BVRB_002280	3	3	187	FOUND	FOUND	FOUND	FOUND	FOUND
A0A0J8E6X7	BVRB_3g067510	12	12	558	FOUND	FOUND	FOUND	FOUND	FOUND
A0A0J8BPV6	BVRB_9g208880	8	7	319	FOUND	FOUND	FOUND	FOUND	FOUND
A0A0J8B746	BVRB_004920	9	9	379	FOUND	FOUND	FOUND	FOUND	FOUND
A0A0J8BBX9	BVRB_5g126360	15	15	534	FOUND	FOUND	FOUND	FOUND	FOUND
A0A023ZQ96	ndhF	12	12	743	FOUND	FOUND	FOUND	FOUND	FOUND
A0A0J8CI10	BVRB_5g105780	7	6	207	FOUND	FOUND	FOUND	FOUND	FOUND
A0A0J8F9V0	BVRB_4g091100	8	8	410	FOUND	FOUND	FOUND	FOUND	FOUND
A0A0J8CFU5	BVRB_4g090850	8	8	347	FOUND	FOUND	FOUND	FOUND	FOUND
A0A0J8E0G2	BVRB_8g201860	4	4	230	FOUND	FOUND	FOUND	FOUND	FOUND
A0A0J8DX81	BVRB_007960	6	5	202	FOUND	FOUND	FOUND	FOUND	FOUND
A0A0J8BBN6	BVRB_5g127130	8	8	718	FOUND	FOUND	FOUND	FOUND	FOUND
A0A0J8BLI2	BVRB_9g206520	9	9	402	FOUND	FOUND	FOUND	FOUND	FOUND
A0A0J8BHA1	BVRB_2g045070	7	7	283	FOUND	FOUND	FOUND	FOUND	FOUND
A0A0J8BD45	BVRB_2g046330	7	7	324	FOUND	FOUND	FOUND	FOUND	FOUND
A0A0J8BES3	BVRB_2g045600	7	7	300	FOUND	FOUND	FOUND	FOUND	FOUND
A0A0J8E5V8	BVRB_3g070690	4	4	147	FOUND	FOUND	FOUND	FOUND	FOUND
A0A0J8CHP4	BVRB_4g087060	7	7	831	FOUND	FOUND	FOUND	FOUND	FOUND
A0A0J8B9T6	BVRB_8g201190	7	7	289	FOUND	FOUND	FOUND	FOUND	FOUND
A0A0J8BG04	BVRB_3g067290	4	4	348	FOUND	FOUND	FOUND	FOUND	FOUND
A0A0J8F6Z5	BVRB_5g105670	3	3	200	FOUND	FOUND	FOUND	FOUND	FOUND
A0A0J8B489	BVRB_016660	1	1	75	FOUND	FOUND	FOUND	FOUND	FOUND
A0A0J8FEI6	BVRB_4g076000	4	4	187	FOUND	FOUND	FOUND	FOUND	FOUND
A0A0J8BDJ3	BVRB_4g095210	5	5	200	FOUND	FOUND	FOUND	FOUND	FOUND
A0A0J8E7U4	BVRB_2g047220	6	6	372	FOUND	FOUND	FOUND	FOUND	FOUND
A0A0J8B2R5	BVRB_009640	4	4	210	FOUND	FOUND	FOUND	FOUND	FOUND
A0A0J8B5T2	BVRB_011680	3	3	216	FOUND	FOUND	FOUND	FOUND	FOUND
A0A0J8B3B3	GATB BVRB_006570	12	12	549	FOUND	FOUND	FOUND	FOUND	FOUND
A0A023ZQA9	rps15	2	2	90	FOUND	FOUND	FOUND	FOUND	FOUND
A0A0J8B944	BVRB_9g225770	3	3	171	FOUND	FOUND	FOUND	FOUND	FOUND
A0A0J8BD98	BVRB_4g097290	10	10	456	FOUND	FOUND	FOUND	FOUND	FOUND
A0A0J8B5L8	BVRB_011380	8	8	264	FOUND	FOUND	FOUND	FOUND	FOUND
A0A0J8BM46	BVRB_9g206040	6	6	339	FOUND	FOUND	FOUND	FOUND	FOUND
A0A0J8ER90	BVRB_7g168190	3	3	153	FOUND	FOUND	FOUND	FOUND	FOUND

Table S1. Table of all identified proteins

A0A023ZQ84	rpl22	7	7	197	FOUND	FOUND	FOUND	FOUND	FOUND
A0A0J8E2P5	BVRB_6g155750	8	8	360	FOUND	FOUND	FOUND	FOUND	FOUND
A0A0J8B3Q2	BVRB_006600	9	9	513	FOUND	FOUND	FOUND	FOUND	FOUND
A0A0J8B878	BVRB_5g126690	9	9	255	FOUND	FOUND	FOUND	FOUND	FOUND
A0A0J8BDS6	BVRB_4g095580	3	3	176	FOUND	FOUND	FOUND	FOUND	FOUND
A0A0J8CKT8	BVRB_4g091120	13	13	1366	FOUND	FOUND	FOUND	FOUND	FOUND
A0A0J8B3C9	BVRB_007680	7	7	518	FOUND	FOUND	FOUND	FOUND	FOUND
A0A0J8FP47	BVRB_2g036800	3	3	228	FOUND	FOUND	FOUND	FOUND	FOUND
Q39440		6	6	281	FOUND	FOUND	FOUND	FOUND	FOUND
A0A0J8B1C6	BVRB_015080	5	5	274	FOUND	FOUND	FOUND	FOUND	FOUND
A0A0J8BCL6	BVRB_3g070190	7	7	503	FOUND	FOUND	FOUND	FOUND	FOUND
A0A0J8BCC1	BVRB_5g124330	7	7	186	FOUND	FOUND	FOUND	FOUND	FOUND
A0A0J8BQQ3	BVRB_9g206230	12	12	536	FOUND	FOUND	FOUND	FOUND	FOUND
A0A0J8CV41	BVRB_2g036880	9	9	223	FOUND	FOUND	FOUND	FOUND	FOUND
A0A0J8E998	BVRB_1g021630	2	2	154	FOUND	FOUND	FOUND	FOUND	FOUND
A0A0J8B787	BVRB_7g178860	2	2	311	FOUND	FOUND	FOUND	FOUND	FOUND
A0A0J8DZ59	BVRB_001600	4	4	177	FOUND	FOUND	FOUND	FOUND	FOUND
A0A0J8B8Q3	BVRB_6g155940	6	6	338	FOUND	FOUND	FOUND	FOUND	FOUND
A0A0J8B8K6	BVRB_000210	4	4	209	FOUND	FOUND	FOUND	FOUND	FOUND
A0A0J8B832	BVRB_003600	1	1	927	FOUND	FOUND	FOUND	NOT FOUND	FOUND
A0A0J8BDE2	BVRB_2g044890	4	4	521	FOUND	FOUND	FOUND	FOUND	FOUND
A0A0J8DZ88	BVRB_001000	9	9	396	FOUND	FOUND	FOUND	FOUND	FOUND
A0A023ZQR1	rps2	4	4	236	FOUND	FOUND	FOUND	FOUND	FOUND
A0A0J8B4N3	BVRB_003480	7	7	1642	FOUND	FOUND	FOUND	FOUND	FOUND
A0A0J8B2B2	BVRB_010610	4	4	209	FOUND	FOUND	FOUND	FOUND	FOUND
A0A0J8CZN0	BVRB_2g036950	2	2	679	FOUND	FOUND	FOUND	FOUND	FOUND
A0A0J8BH96	BVRB_2g045020	9	9	322	FOUND	FOUND	FOUND	FOUND	FOUND
A0A023ZPU1	rpl20	2	2	128	FOUND	FOUND	FOUND	FOUND	FOUND
A0A0J8B6R4	BVRB_006660	4	4	291	FOUND	FOUND	FOUND	FOUND	FOUND
A0A0J8FG78	BVRB_3g065550	4	4	154	FOUND	FOUND	FOUND	FOUND	FOUND
A0A0J8E1V0	BVRB_7g179310	5	5	204	FOUND	FOUND	FOUND	FOUND	FOUND
A0A0J8B3A6	BVRB_006690	4	4	312	FOUND	FOUND	FOUND	FOUND	FOUND
A0A0J8BH75	BVRB_2g044830	8	8	571	FOUND	FOUND	FOUND	FOUND	FOUND
A0A0J8BHG7	BVRB_2g044520	6	6	274	FOUND	FOUND	FOUND	FOUND	FOUND
A0A023ZQ47	ndhK nuoB	3	3	227	FOUND	FOUND	FOUND	FOUND	FOUND
A0A023ZRC0	rps18	3	3	101	FOUND	FOUND	FOUND	FOUND	FOUND
A0A0J8CRX9	BVRB_3g065250	1	1	283	FOUND	FOUND	FOUND	FOUND	FOUND
A0A0J8B523	BVRB_002560	7	7	1041	FOUND	FOUND	FOUND	FOUND	FOUND
A0A0J8B2U4	BVRB_008180	12	12	820	FOUND	FOUND	FOUND	FOUND	FOUND
A0A0J8CBM6	BVRB_5g112650	1	1	140	FOUND	FOUND	FOUND	FOUND	FOUND
A0A0J8B538	BVRB_013480	5	5	349	FOUND	FOUND	FOUND	FOUND	FOUND
A0A0J8E380	BVRB_5g126230	3	3	432	FOUND	FOUND	FOUND	FOUND	FOUND
A0A0J8C3V6	BVRB_6g140250	5	5	297	FOUND	FOUND	FOUND	FOUND	FOUND
A0A0J8BGA9	BVRB_042230	3	3	126	FOUND	FOUND	FOUND	FOUND	FOUND
A0A0J8B5D6	BVRB_012510	3	3	135	FOUND	FOUND	FOUND	FOUND	FOUND

Table S1. Table of all identified proteins

A0A0J8C0I0	BVRB_7g165930	8	8	632	FOUND	FOUND	FOUND	FOUND	FOUND
A0A0J8BQN8	BVRB_9g206170	1	1	729	FOUND	FOUND	FOUND	FOUND	FOUND
A0A0J8B812	BVRB_6g155420	12	12	792	FOUND	FOUND	FOUND	FOUND	FOUND
A0A0J8BAD6	BVRB_5g123200	6	6	212	FOUND	FOUND	FOUND	FOUND	FOUND
A0A0J8AWX7	BVRB_032230	5	1	126	FOUND	FOUND	FOUND	FOUND	FOUND
A0A0J8D1W5	BVRB_2g031820	5	5	206	FOUND	FOUND	FOUND	FOUND	FOUND
A0A0J8FG96	BVRB_3g065740	11	11	873	FOUND	FOUND	FOUND	FOUND	FOUND
A0A0J8B8Q7	BVRB_6g155970	6	6	324	FOUND	FOUND	FOUND	FOUND	FOUND
A0A0J8B767	BVRB_006430	10	10	688	FOUND	FOUND	FOUND	FOUND	FOUND
A0A023ZPS0	ndhJ nuoC	7	7	158	FOUND	FOUND	FOUND	FOUND	FOUND
A0A0J8B770	BVRB_7g178660	9	9	472	FOUND	FOUND	FOUND	FOUND	FOUND
A0A0J8BDC6	BVRB_4g096100	5	1	219	FOUND	FOUND	FOUND	FOUND	FOUND
A0A0J8B9N7	BVRB_4g096810	15	15	734	FOUND	FOUND	FOUND	FOUND	FOUND
A0A0J8DW07	BVRB_013170	7	7	337	FOUND	FOUND	FOUND	FOUND	FOUND
A0A0J8EXZ5	BVRB_6g143910	5	5	351	FOUND	FOUND	FOUND	FOUND	FOUND
A0A0J8BAW1	BVRB_7g178840	5	5	461	FOUND	FOUND	FOUND	FOUND	FOUND
A0A0J8B593	BVRB_013130	5	2	298	FOUND	FOUND	FOUND	FOUND	FOUND
Q0QG29	cox2	2	2	260	FOUND	FOUND	FOUND	FOUND	FOUND
A0A0J8B9W4	BVRB_8g199660	5	5	310	FOUND	FOUND	FOUND	FOUND	FOUND
A0A0J8BPU7	BVRB_9g208920	4	4	217	FOUND	FOUND	FOUND	FOUND	FOUND
A0A0J8B4M5	BVRB_015280	2	2	177	FOUND	FOUND	FOUND	FOUND	FOUND
A0A0J8CMT7	BVRB_4g084490	4	4	178	FOUND	FOUND	FOUND	FOUND	FOUND
A0A0J8B4I1	BVRB_015520	2	2	76	FOUND	FOUND	FOUND	FOUND	FOUND
A0A0J8B4G9	BVRB_002340	7	7	435	FOUND	FOUND	FOUND	FOUND	FOUND
A0A023ZRF6	ndhA nuoH	4	4	363	FOUND	FOUND	FOUND	FOUND	FOUND
A0A0J8CB59	BVRB_5g113530	7	7	316	FOUND	FOUND	FOUND	FOUND	FOUND
A0A0J8E591	BVRB_4g093420	5	5	419	FOUND	FOUND	FOUND	FOUND	FOUND
Q9MFD6	orf25	3	3	198	FOUND	FOUND	FOUND	FOUND	FOUND
A0A0J8B6M6	BVRB_008210	7	7	306	FOUND	FOUND	FOUND	FOUND	FOUND
Q5U6B5	atp8	5	5	217	FOUND	FOUND	FOUND	FOUND	FOUND
A0A0J8B9C6	BVRB_8g201530	7	7	125	FOUND	FOUND	FOUND	FOUND	FOUND
A0A0J8B2H4	BVRB_009360	6	6	301	FOUND	FOUND	FOUND	FOUND	FOUND
A0A0J8E3S1	BVRB_5g124030	2	2	146	FOUND	FOUND	FOUND	FOUND	FOUND
A0A0J8E105	BVRB_8g199680	6	6	325	FOUND	FOUND	FOUND	FOUND	FOUND
A0A0J8BCW5	BVRB_2g047210	6	6	390	FOUND	FOUND	FOUND	FOUND	FOUND
A0A0J8BDS4	BVRB_4g094530	11	11	555	FOUND	FOUND	FOUND	FOUND	FOUND
A0A0J8BDF3	BVRB_3g067270	5	5	332	FOUND	FOUND	FOUND	FOUND	FOUND
A0A0J8BHD9	BVRB_2g045430	7	7	285	FOUND	FOUND	FOUND	FOUND	FOUND
A0A0J8FQD3	BVRB_2g033270	10	10	531	FOUND	FOUND	FOUND	FOUND	FOUND
Q9SPH6	pt	2	2	277	FOUND	FOUND	FOUND	FOUND	FOUND
A0A0J8B6K7	BVRB_008350	2	2	225	FOUND	FOUND	FOUND	FOUND	FOUND
A0A0J8E2Q9	BVRB_6g155590	9	9	578	FOUND	FOUND	FOUND	FOUND	FOUND
A0A0J8E8A5	BVRB_2g044900	13	6	740	FOUND	FOUND	FOUND	FOUND	FOUND
A0A0J8BLK4	BVRB_9g206080	7	7	574	FOUND	FOUND	FOUND	FOUND	FOUND
A0A0J8B4W7	BVRB_014340	5	5	149	FOUND	FOUND	FOUND	FOUND	FOUND

Table S1. Table of all identified proteins

A0A0J8B312	BVRB_023380	1	1	180	FOUND	FOUND	FOUND	FOUND	FOUND
A0A0J8B464	BVRB_004860	2	2	439	FOUND	FOUND	FOUND	FOUND	FOUND
A0A0J8B6Q9	BVRB_8g200960	4	4	780	FOUND	FOUND	FOUND	FOUND	FOUND
A0A0J8B1T0	BVRB_027290	2	2	181	FOUND	FOUND	FOUND	FOUND	FOUND
A0A0J8B999	BVRB_5g123230	1	1	1849	FOUND	FOUND	FOUND	FOUND	FOUND
A0A0J8BAA8	BVRB_5g122960	5	5	186	FOUND	FOUND	FOUND	FOUND	FOUND
A0A0J8BBZ0	BVRB_5g125990	5	5	239	FOUND	FOUND	FOUND	FOUND	FOUND
A0A0J8DXQ5	BVRB_006300	4	4	255	FOUND	FOUND	FOUND	FOUND	FOUND
A0A0J8B2S4	BVRB_008370	7	7	496	FOUND	FOUND	FOUND	FOUND	FOUND
A0A0J8E5W2	BVRB_3g070740	4	4	144	FOUND	FOUND	FOUND	FOUND	FOUND
A0A0J8C0U2	BVRB_7g165090	4	4	161	FOUND	FOUND	FOUND	FOUND	FOUND
A0A0J8DW49	BVRB_012960	4	4	350	FOUND	FOUND	FOUND	FOUND	FOUND
A0A0J8BV83	BVRB_8g187720	8	8	781	FOUND	FOUND	FOUND	FOUND	FOUND
A0A0J8B3J3	BVRB_007300	1	1	424	FOUND	FOUND	FOUND	FOUND	FOUND
A0A0J8B5A3	BVRB_9g225390	3	3	315	FOUND	FOUND	FOUND	FOUND	FOUND
A0A0J8D1H6	BVRB_2g031880	1	1	1050	NOT FOUND	NOT FOUND	FOUND	FOUND	NOT FOUND
A0A0J8B454	BVRB_005030	10	10	994	FOUND	FOUND	FOUND	FOUND	FOUND
A0A0J8E4I4	BVRB_4g096520	9	9	539	FOUND	FOUND	FOUND	FOUND	FOUND
A0A0J8FG47	BVRB_3g065270	8	8	345	FOUND	FOUND	FOUND	FOUND	FOUND
A0A0J8EGU5	BVRB_9g207010	9	9	910	FOUND	FOUND	FOUND	FOUND	FOUND
A0A0J8CBL1	BVRB_5g112600	5	5	1041	FOUND	FOUND	FOUND	FOUND	FOUND
A0A0J8CZN6	BVRB_2g036650	4	4	136	FOUND	FOUND	FOUND	FOUND	FOUND
A0A0J8F9W0	BVRB_4g091190	1	1	1809	NOT FOUND	FOUND	NOT FOUND	NOT FOUND	FOUND
A0A0J8B792	BVRB_006320	3	3	204	FOUND	FOUND	FOUND	FOUND	FOUND
A0A0J8B9R3	BVRB_4g096550	3	3	292	FOUND	FOUND	FOUND	FOUND	FOUND
A0A0J8CX02	BVRB_3g051010	3	3	300	FOUND	FOUND	FOUND	FOUND	FOUND
A0A0J8B3M8	BVRB_005270	5	5	229	FOUND	FOUND	FOUND	FOUND	FOUND
A0A0J8B7B7	BVRB_005950	1	1	130	FOUND	FOUND	FOUND	FOUND	FOUND
A0A0J8B375	BVRB_007100	4	4	510	FOUND	FOUND	FOUND	FOUND	FOUND
A0A0J8B761	BVRB_7g178560	1	1	341	FOUND	FOUND	FOUND	FOUND	FOUND
A0A0J8B714	BVRB_005260	9	9	553	FOUND	FOUND	FOUND	FOUND	FOUND
A0A0J8BAU3	BVRB_4g096480	3	3	225	FOUND	FOUND	FOUND	FOUND	FOUND
A0A0J8BLG5	BVRB_9g209050	2	2	237	FOUND	FOUND	FOUND	FOUND	FOUND
A0A0J8FC93	BVRB_4g083570	1	1	139	FOUND	FOUND	FOUND	FOUND	FOUND
A0A0J8DYB3	BVRB_004250	1	1	78	FOUND	FOUND	FOUND	FOUND	FOUND
A0A0J8B4X3	BVRB_014130	1	1	336	FOUND	FOUND	FOUND	FOUND	FOUND
A0A0J8B8A3	BVRB_001030	8	8	397	FOUND	FOUND	FOUND	FOUND	FOUND
A0A7G2RM36	BVRB_8g200620	5	5	273	FOUND	FOUND	FOUND	FOUND	FOUND
A0A0J8E2Y3	BVRB_5g126870	8	8	621	FOUND	FOUND	FOUND	FOUND	FOUND
A0A0J8B3K4	BVRB_005660	3	3	265	FOUND	FOUND	FOUND	FOUND	FOUND
A0A0J8B9G9	BVRB_9g224400	6	6	489	FOUND	FOUND	FOUND	FOUND	FOUND
A0A0J8BW11	BVRB_8g184840	6	6	340	FOUND	FOUND	FOUND	FOUND	FOUND
A0A0J8CQI2	BVRB_4g076250	6	6	1096	FOUND	FOUND	FOUND	FOUND	FOUND
A0A0J8BCS5	BVRB_2g047520	1	1	110	FOUND	FOUND	FOUND	FOUND	FOUND
A0A0J8BEC0	BVRB_2g046300	4	4	88	FOUND	FOUND	FOUND	FOUND	FOUND

Table S1. Table of all identified proteins

A0A0J8BHP8	BVRB_1g022990	10	10	561	FOUND	FOUND	FOUND	FOUND	FOUND
A0A0J8D1C1	BVRB_2g033020	1	1	567	FOUND	FOUND	FOUND	FOUND	FOUND
A0A0J8CPI6	BVRB_3g059870	7	7	422	FOUND	FOUND	FOUND	FOUND	FOUND
A0A0J8B7X0	BVRB_004020	3	3	396	FOUND	FOUND	FOUND	FOUND	FOUND
A0A0J8BBC3	BVRB_4g095130	5	5	120	FOUND	FOUND	FOUND	FOUND	FOUND
A0A0J8B4Z9	BVRB_014180	6	6	381	FOUND	FOUND	FOUND	FOUND	FOUND
A0A0J8B352	BVRB_008340	8	8	693	FOUND	FOUND	FOUND	FOUND	FOUND
A0A7G2RLY6	BVRB_8g200140	3	3	332	FOUND	NOT FOUND	FOUND	FOUND	FOUND
A0A0J8BEX1	BVRB_1g023310	1	1	145	FOUND	FOUND	FOUND	FOUND	FOUND
A0A0J8DY30	BVRB_005070	5	5	487	FOUND	FOUND	FOUND	FOUND	FOUND
A0A0J8B2F7	BVRB_009540	3	3	143	FOUND	FOUND	FOUND	FOUND	FOUND
A0A0J8BD15	BVRB_4g096820	5	5	293	FOUND	FOUND	FOUND	FOUND	FOUND
A0A0J8B227	BVRB_012180	2	2	348	FOUND	FOUND	FOUND	FOUND	FOUND
A0A0J8B2Q3	BVRB_008570	5	5	420	FOUND	FOUND	FOUND	FOUND	FOUND
A0A0J8B365	BVRB_007170	6	6	626	FOUND	FOUND	FOUND	FOUND	FOUND
A0A0J8CW87	BVRB_2g033260	3	3	203	FOUND	FOUND	FOUND	FOUND	FOUND
A0A0J8CKI9	BVRB_4g076160	3	3	263	FOUND	FOUND	FOUND	FOUND	FOUND
A0A0J8B4J3	BVRB_002160	5	5	350	FOUND	FOUND	FOUND	FOUND	FOUND
A0A0J8E8E0	BVRB_2g045330	1	1	71	FOUND	FOUND	FOUND	FOUND	FOUND
A0A0J8BAE9	BVRB_4g097520	4	4	219	FOUND	FOUND	FOUND	FOUND	FOUND
A0A0J8B9B6	BVRB_4g097600	5	5	127	FOUND	FOUND	FOUND	FOUND	FOUND
A0A0J8DZL2	BVRB_000230	5	5	237	FOUND	FOUND	FOUND	FOUND	FOUND
A0A0J8E1N5	BVRB_7g179490	5	5	254	FOUND	FOUND	FOUND	FOUND	FOUND
A0A0J8D0Z9	BVRB_2g033350	3	3	212	FOUND	FOUND	FOUND	FOUND	FOUND
A0A0J8DSQ2	BVRB_028030	3	5	167	FOUND	FOUND	FOUND	FOUND	FOUND
A0A0J8DWR3	BVRB_009550	1	1	443	FOUND	FOUND	FOUND	FOUND	FOUND
A0A0J8BCI1	BVRB_5g124770	2	2	451	FOUND	FOUND	FOUND	FOUND	FOUND
A0A0J8CU05	BVRB_3g059830	6	6	361	FOUND	FOUND	FOUND	FOUND	FOUND
O81266		2	2	84	FOUND	FOUND	FOUND	FOUND	FOUND
A0A0J8E6Y2	BVRB_3g067550	4	4	1079	FOUND	FOUND	FOUND	FOUND	FOUND
A0A0J8B963	BVRB_9g225520	3	3	285	FOUND	FOUND	FOUND	FOUND	FOUND
A0A0J8B6M8	BVRB_007090	5	5	635	FOUND	FOUND	FOUND	FOUND	FOUND
A0A0J8B402	BVRB_003970	3	3	858	FOUND	FOUND	FOUND	FOUND	FOUND
A0A0J8BHN8	BVRB_1g023210	5	1	302	FOUND	FOUND	FOUND	FOUND	FOUND
A0A0J8C149	BVRB_7g164940	4	4	199	FOUND	FOUND	FOUND	FOUND	FOUND
A0A7G2RM29	BVRB_8g200490	3	3	414	FOUND	FOUND	FOUND	FOUND	FOUND
A0A0J8B6I7	BVRB_8g201360	4	4	268	FOUND	FOUND	FOUND	FOUND	FOUND
A0A0J8EGX4	BVRB_9g206390	1	1	116	FOUND	FOUND	FOUND	FOUND	FOUND
A0A0J8CIG0	BVRB_5g104510	4	4	321	FOUND	FOUND	FOUND	FOUND	FOUND
A0A0J8BW84	BVRB_8g184830	4	4	270	FOUND	FOUND	FOUND	FOUND	FOUND
A0A0J8DXP0	BVRB_006410	4	4	225	FOUND	FOUND	FOUND	FOUND	FOUND
A0A0J8BBB6	BVRB_6g156440 BVRB_6g156470	3	3	86	FOUND	FOUND	FOUND	FOUND	FOUND
A0A0J8B9S9	BVRB_8g201420	5	5	253	FOUND	FOUND	FOUND	FOUND	FOUND
Q0QG26	nad1	4	4	325	FOUND	FOUND	FOUND	FOUND	FOUND
Q9MF98	cob	2	2	393	FOUND	NOT FOUND	FOUND	FOUND	FOUND

Table S1. Table of all identified proteins

A0A0J8E100	BVRB_8g199630	1	1	423	FOUND	FOUND	FOUND	FOUND	FOUND
A0A0J8FGB3	BVRB_3g065950	1	1	379	FOUND	FOUND	FOUND	FOUND	FOUND
A0A0J8BBJ2	BVRB_6g156160	5	5	442	FOUND	FOUND	FOUND	FOUND	FOUND
A0A0J8B5D5	BVRB_001210	4	4	208	FOUND	FOUND	FOUND	FOUND	FOUND
A0A0J8B7X8	BVRB_002480	3	3	128	FOUND	FOUND	FOUND	FOUND	FOUND
A0A0J8EGU0	BVRB_9g206980	7	7	767	FOUND	FOUND	FOUND	FOUND	FOUND
A0A0J8ELP0	BVRB_8g187700	4	4	709	FOUND	FOUND	FOUND	FOUND	FOUND
A0A0J8DVD8	BVRB_015110	1	1	88	FOUND	FOUND	FOUND	FOUND	FOUND
A0A0J8BCV9	BVRB_4g097590	4	4	161	FOUND	FOUND	FOUND	FOUND	FOUND
A0A0J8CQL5	BVRB_3g055650	5	5	291	FOUND	FOUND	FOUND	FOUND	FOUND
A0A0J8CKR1	BVRB_4g091210	4	4	230	FOUND	FOUND	FOUND	FOUND	FOUND
A0A0J8CWK4	BVRB_2g033330	2	2	437	FOUND	FOUND	FOUND	FOUND	FOUND
A0A0J8E8Y0	BVRB_1g022780	4	4	445	FOUND	FOUND	FOUND	FOUND	FOUND
A0A0J8BCX9	BVRB_2g046980	4	4	573	FOUND	FOUND	FOUND	FOUND	FOUND
A0A0J8BHK5	BVRB_1g023200	1	1	123	FOUND	FOUND	FOUND	FOUND	FOUND
A0A0J8B8L9	BVRB_001570	3	3	363	FOUND	FOUND	FOUND	FOUND	FOUND
A0A0J8E049	BVRB_9g224680	3	3	513	FOUND	FOUND	FOUND	FOUND	FOUND
A0A0N9LMF7	ycf3	2	2	126	FOUND	FOUND	FOUND	FOUND	FOUND
A0A0J8EH07	BVRB_9g206090	6	6	731	FOUND	FOUND	FOUND	FOUND	FOUND
A0A0J8BF45	BVRB_3g070550	1	1	167	FOUND	NOT FOUND	NOT FOUND	NOT FOUND	NOT FOUND
A0A0J8BPT2	BVRB_9g209020	1	1	78	FOUND	FOUND	FOUND	FOUND	FOUND
A0A0J8E8D7	BVRB_2g045280	2	2	192	FOUND	FOUND	FOUND	FOUND	FOUND
A0A0J8B3I5	BVRB_005760	4	4	856	FOUND	FOUND	FOUND	FOUND	FOUND
A0A0J8DY36	BVRB_004840	3	3	114	FOUND	FOUND	FOUND	FOUND	FOUND
A0A0J8B5I8	BVRB_012760	1	1	79	FOUND	FOUND	FOUND	FOUND	FOUND
A0A0J8BGL1	BVRB_2g047070	1	1	305	FOUND	FOUND	FOUND	FOUND	FOUND
A0A0J8CQE4	BVRB_4g075800	4	4	342	FOUND	FOUND	FOUND	FOUND	FOUND
Q9MF62	nad4	2	2	495	FOUND	NOT FOUND	NOT FOUND	FOUND	FOUND
A0A0J8B9J6	BVRB_9g224430	2	2	217	FOUND	FOUND	FOUND	FOUND	FOUND
A0A0J8E2K4	BVRB_6g156140	5	5	587	FOUND	FOUND	FOUND	FOUND	FOUND
A0A7G2RMG2	BVRB_8g200630	4	4	224	FOUND	FOUND	FOUND	FOUND	FOUND
A0A0J8AXW9	BVRB_029050	1	1	138	NOT FOUND	FOUND	FOUND	FOUND	FOUND
A0A023ZQ52	accD	3	3	524	FOUND	FOUND	FOUND	FOUND	FOUND
A0A0J8CS48	BVRB_3g065290	5	5	626	FOUND	FOUND	FOUND	FOUND	FOUND
A0A0J8F5E4	BVRB_5g111290	2	2	211	FOUND	FOUND	FOUND	FOUND	FOUND
A0A0J8CWD5	BVRB_3g050380	1	1	245	FOUND	FOUND	FOUND	FOUND	FOUND
A0A0J8BAP2	BVRB_7g179440	3	3	235	FOUND	FOUND	FOUND	FOUND	FOUND
A0A0J8BFU1	BVRB_3g067760	1	1	315	FOUND	FOUND	FOUND	FOUND	FOUND
A0A0J8B4L3	BVRB_001700	2	2	104	FOUND	FOUND	FOUND	FOUND	FOUND
A0A0J8DVB5	BVRB_015320	1	1	244	FOUND	FOUND	FOUND	NOT FOUND	FOUND
A0A023ZQX0	infA	2	2	77	FOUND	FOUND	FOUND	FOUND	FOUND
A0A0J8B5K7	BVRB_011480	2	2	200	FOUND	FOUND	FOUND	FOUND	FOUND
A0A0J8CI46	BVRB_5g105720	1	1	70	FOUND	FOUND	FOUND	FOUND	FOUND
A0A0J8CI11	BVRB_4g084410	3	3	559	FOUND	FOUND	FOUND	FOUND	FOUND
A0A0J8BC25	BVRB_5g126580	1	1	247	FOUND	FOUND	FOUND	FOUND	FOUND

Table S1. Table of all identified proteins

A0A0J8CTR3	BVRB_3g059800	6	6	369	FOUND	FOUND	FOUND	FOUND	FOUND
A0A0J8DWD6	BVRB_011670	3	3	187	FOUND	FOUND	FOUND	FOUND	FOUND
A0A0J8B7R3	BVRB_004560	1	1	123	FOUND	FOUND	NOT FOUND	NOT FOUND	FOUND
A0A0J8BH48	BVRB_2g045620	3	3	594	FOUND	FOUND	FOUND	FOUND	FOUND
A0A023ZQB9	ycf4	2	2	184	FOUND	FOUND	FOUND	FOUND	FOUND
A0A0J8B807	MENG BVRB_6g155480	3	3	265	FOUND	FOUND	FOUND	FOUND	FOUND
A0A0J8EH11	BVRB_9g206140	7	7	405	FOUND	FOUND	FOUND	FOUND	FOUND
A0A0J8E0H9	BVRB_8g201350	3	3	108	FOUND	FOUND	FOUND	FOUND	FOUND
A0A0J8DX51	BVRB_008240	8	8	884	FOUND	FOUND	FOUND	FOUND	FOUND
A0A0J8DVI3	BVRB_014620	4	4	413	FOUND	FOUND	FOUND	FOUND	FOUND
A0A0J8CWP6	BVRB_2g031890	4	4	463	FOUND	FOUND	FOUND	FOUND	FOUND
A0A0J8B131	BVRB_016190	1	1	209	FOUND	FOUND	FOUND	FOUND	FOUND
A0A0J8D0R0	BVRB_007550	2	2	142	FOUND	FOUND	FOUND	FOUND	FOUND
A0A0J8B3T7	BVRB_006310	4	4	223	FOUND	FOUND	FOUND	FOUND	FOUND
A0A0J8E0P8	BVRB_8g200670	3	3	314	FOUND	FOUND	FOUND	FOUND	FOUND
A0A0J8D1G5	BVRB_2g033340	3	3	633	FOUND	FOUND	FOUND	FOUND	FOUND
A0A0J8B9C1	BVRB_4g097340	1	1	239	FOUND	FOUND	FOUND	FOUND	FOUND
A0A0J8BHP0	BVRB_1g022630	3	3	221	FOUND	FOUND	FOUND	FOUND	FOUND
A0A0J8ES22	BVRB_7g165110	3	3	161	FOUND	FOUND	FOUND	FOUND	FOUND
A0A0J8B293	BVRB_011290	1	1	402	FOUND	FOUND	FOUND	FOUND	FOUND
A0A0J8B2Q1	BVRB_009860	2	2	351	FOUND	NOT FOUND	NOT FOUND	FOUND	FOUND
A0A0J8CN27	BVRB_4g083490	2	2	341	FOUND	FOUND	FOUND	FOUND	FOUND
A0A0J8BGM5	BVRB_2g047030	1	1	211	FOUND	FOUND	FOUND	FOUND	FOUND
Q9MF79	nad2	2	2	488	FOUND	FOUND	FOUND	FOUND	FOUND
A0A0J8B552	BVRB_002030	4	4	388	FOUND	FOUND	FOUND	FOUND	FOUND
A0A0J8E886	BVRB_2g045730	6	6	535	FOUND	FOUND	FOUND	FOUND	FOUND
A0A0J8B981	BVRB_9g225180	1	1	546	FOUND	FOUND	FOUND	FOUND	FOUND
A0A0J8B717	BVRB_8g199790	2	2	209	FOUND	FOUND	FOUND	FOUND	FOUND
A0A0J8E0B8	BVRB_8g202010	1	1	131	FOUND	FOUND	FOUND	FOUND	FOUND
A0A0J8CS33	BVRB_3g065820	5	5	412	FOUND	FOUND	FOUND	FOUND	FOUND
A0A0J8DZT0	BVRB_9g225800	5	5	418	FOUND	FOUND	FOUND	FOUND	FOUND
A0A0J8E6S4	BVRB_3g067950	3	3	328	FOUND	FOUND	FOUND	FOUND	FOUND
A0A0J8F517	BVRB_5g112640	4	4	302	FOUND	FOUND	FOUND	FOUND	FOUND
A0A0J8E8U1	BVRB_1g023230	5	1	302	FOUND	FOUND	FOUND	FOUND	FOUND
A0A0J8B7Q8	BVRB_7g179630	4	4	251	NOT FOUND	FOUND	FOUND	FOUND	FOUND
A0A0J8CDM0	BVRB_5g104560	4	1	138	FOUND	FOUND	FOUND	FOUND	FOUND
A0A0J8BE70	BVRB_2g046990	4	4	548	FOUND	FOUND	FOUND	FOUND	FOUND
A0A0J8CNY8	BVRB_3g059690	2	2	1170	FOUND	FOUND	FOUND	FOUND	FOUND
A0A0J8B5J0	BVRB_000810	1	1	1124	FOUND	FOUND	FOUND	FOUND	FOUND
Q9SM24	Bv8-6	2	2	539	FOUND	NOT FOUND	FOUND	FOUND	FOUND
A0A0J8B6M1	BVRB_7g180480	2	2	214	FOUND	FOUND	FOUND	FOUND	FOUND
A0A0J8B382	BVRB_008080	6	2	214	FOUND	FOUND	FOUND	FOUND	FOUND
A0A0J8B711	BVRB_7g179660	1	1	967	NOT FOUND	FOUND	FOUND	FOUND	NOT FOUND
A0A0J8EYF7	BVRB_6g142310	4	4	398	FOUND	FOUND	FOUND	FOUND	FOUND
A0A0J8BCI6	BVRB_5g124820	1	1	496	FOUND	FOUND	FOUND	FOUND	FOUND

Table S1. Table of all identified proteins

A0A0J8B1Q4	BVRB_027550	5	2	213	FOUND	FOUND	FOUND	NOT FOUND	NOT FOUND
A0A0J8CV33	BVRB_2g036790	4	4	474	FOUND	FOUND	FOUND	FOUND	FOUND
A0A023ZRD1	rpoA	3	3	336	FOUND	FOUND	FOUND	FOUND	FOUND
A0A023ZQ44	rps14	1	1	100	FOUND	FOUND	FOUND	NOT FOUND	FOUND
A0A0J8BEJ9	BVRB_1g020730	3	3	541	FOUND	FOUND	FOUND	FOUND	FOUND
A0A0J8BCR0	BVRB_5g124230	3	3	368	FOUND	FOUND	FOUND	FOUND	FOUND
A0A0J8FQE7	BVRB_2g033410	3	3	365	FOUND	FOUND	FOUND	FOUND	FOUND
A0A0J8E8T6	BVRB_1g023190	1	1	191	FOUND	FOUND	FOUND	FOUND	FOUND
A0A0J8BAT7	BVRB_7g179180	2	2	279	FOUND	FOUND	FOUND	FOUND	FOUND
P93076	BvcDNA-397	1	1	545	FOUND	FOUND	FOUND	FOUND	FOUND
A0A0J8B3G0	BVRB_007670	1	1	127	FOUND	FOUND	FOUND	FOUND	FOUND
A0A0J8B8K1	BVRB_5g125630	1	1	503	FOUND	NOT FOUND	FOUND	NOT FOUND	FOUND
A0A0J8BLH6	BVRB_9g206480	1	1	191	FOUND	FOUND	FOUND	FOUND	FOUND
A0A0J8B4Q2	BVRB_003190	3	3	386	FOUND	FOUND	NOT FOUND	FOUND	FOUND
A0A0J8BA63	BVRB_8g199700	2	2	313	FOUND	FOUND	FOUND	FOUND	FOUND
A0A0J8BET8	BVRB_2g044550	1	1	211	FOUND	FOUND	FOUND	FOUND	FOUND
A0A0J8D0B1	BVRB_2g036940	4	4	250	FOUND	FOUND	FOUND	FOUND	FOUND
A0A0J8BAN2	BVRB_7g179560	4	4	454	FOUND	FOUND	FOUND	FOUND	FOUND
A0A0J8FP32	BVRB_2g037010	3	1	124	FOUND	FOUND	FOUND	FOUND	FOUND
A0A0J8CFG6	BVRB_5g113760	5	5	480	FOUND	FOUND	FOUND	FOUND	NOT FOUND
A0A0J8DX36	BVRB_008250	4	4	685	FOUND	FOUND	NOT FOUND	FOUND	FOUND
A0A0J8BME3	BVRB_9g204780	15	4	421	FOUND	FOUND	FOUND	FOUND	FOUND
A0A0J8B833	BVRB_002200	1	1	223	FOUND	FOUND	NOT FOUND	FOUND	FOUND
A0A0J8DXP3	BVRB_006460	1	1	778	FOUND	NOT FOUND	FOUND	FOUND	NOT FOUND
A0A0J8B3B5	BVRB_006620	2	2	360	FOUND	FOUND	FOUND	NOT FOUND	FOUND
A0A0J8B782	BVRB_004830	3	3	302	FOUND	FOUND	FOUND	FOUND	FOUND
A0A0J8CWV6	BVRB_3g050660	2	2	427	FOUND	FOUND	FOUND	FOUND	FOUND
A0A0J8CZ41	BVRB_2g047340	1	1	51	FOUND	FOUND	FOUND	FOUND	FOUND
A0A0J8B4E6	BVRB_002720	4	4	504	FOUND	FOUND	FOUND	FOUND	FOUND
A0A0J8B705	BVRB_005450	2	2	250	FOUND	FOUND	FOUND	FOUND	FOUND
A0A0J8FQB8	BVRB_2g033120	2	1	488	FOUND	FOUND	NOT FOUND	FOUND	FOUND
Q9MF46	nad5	2	2	670	NOT FOUND	NOT FOUND	NOT FOUND	FOUND	FOUND
A0A0J8DWP7	BVRB_009630	1	1	233	FOUND	FOUND	NOT FOUND	FOUND	FOUND
A0A0J8BDA5	BVRB_4g096540	3	3	371	FOUND	FOUND	FOUND	FOUND	FOUND
A0A0J8BLI0	BVRB_9g208860	1	1	320	NOT FOUND	FOUND	FOUND	NOT FOUND	FOUND
A0A023ZRF1	ndhD	2	2	500	FOUND	FOUND	FOUND	FOUND	FOUND
A0A0J8BD72	BVRB_4g097630	1	1	233	FOUND	FOUND	NOT FOUND	FOUND	FOUND
A0A0J8CWI9	BVRB_2g033180	2	2	473	FOUND	FOUND	NOT FOUND	FOUND	NOT FOUND
A0A0J8B3T4	BVRB_004400	2	2	230	FOUND	FOUND	FOUND	FOUND	FOUND
A0A0J8FEF7	BVRB_4g075730	4	4	887	FOUND	FOUND	FOUND	FOUND	FOUND
A0A0J8E8M2	BVRB_2g044730	1	1	836	FOUND	FOUND	FOUND	FOUND	FOUND
A0A0J8B9L8	BVRB_8g202030	1	1	459	FOUND	FOUND	NOT FOUND	NOT FOUND	NOT FOUND
A0A0J8B5N9	BVRB_012090	1	1	148	FOUND	FOUND	FOUND	FOUND	FOUND
A0A0J8E8N3	BVRB_1g023290	1	1	489	FOUND	FOUND	FOUND	FOUND	FOUND
A0A0J8B9H8	BVRB_4g096830	1	1	207	NOT FOUND	FOUND	FOUND	NOT FOUND	FOUND

Table S1. Table of all identified proteins

A0A0J8CFS9	BVRB_5g112690	4	4	861	FOUND	FOUND	FOUND	FOUND	FOUND
A0A0J8B9A8	BVRB_9g224960	1	1	189	FOUND	NOT FOUND	NOT FOUND	FOUND	FOUND
A0A0J8BF57	BVRB_019750	2	2	153	FOUND	FOUND	NOT FOUND	FOUND	FOUND
A0A0J8BGK6	BVRB_2g047130	2	2	360	FOUND	NOT FOUND	FOUND	FOUND	FOUND
A0A0J8B8C4	BVRB_002700	2	2	368	FOUND	FOUND	FOUND	FOUND	FOUND
A0A0J8BB69	BVRB_7g177590	3	3	402	FOUND	FOUND	FOUND	FOUND	FOUND
A0A0J8FQT7	BVRB_2g031750	1	1	94	NOT FOUND	FOUND	NOT FOUND	FOUND	NOT FOUND
A0A023ZR01	ycf1	6	6	1848	FOUND	FOUND	FOUND	FOUND	FOUND
A0A0J8CWJ9	BVRB_2g033280	2	2	260	FOUND	FOUND	FOUND	FOUND	FOUND
A0A0J8B522	BVRB_013630	1	1	215	FOUND	FOUND	FOUND	FOUND	FOUND
A0A0J8BB62	BVRB_4g095660	1	1	444	FOUND	FOUND	FOUND	FOUND	FOUND
A0A0J8B9T8	BVRB_4g096160	1	1	154	NOT FOUND	NOT FOUND	FOUND	FOUND	NOT FOUND
A0A0J8BAS8	BVRB_4g096630	2	2	408	NOT FOUND	FOUND	FOUND	FOUND	FOUND
A0A0J8DZW6	BVRB_9g225470	2	2	199	FOUND	FOUND	FOUND	FOUND	FOUND
A0A0J8B640	BVRB_009890	1	1	523	FOUND	FOUND	FOUND	FOUND	FOUND
A0A0J8BVG7	BVRB_7g168320	1	1	228	NOT FOUND	NOT FOUND	FOUND	NOT FOUND	FOUND
A0A0J8BB81	BVRB_3g070320	1	1	1060	NOT FOUND	FOUND	FOUND	FOUND	NOT FOUND
A0A0J8B4D7	BVRB_002630	2	2	135	FOUND	FOUND	FOUND	FOUND	FOUND
J3S7K8		1	1	155	FOUND	FOUND	FOUND	FOUND	FOUND
A0A0J8B5N7	BVRB_8g202000	2	2	389	FOUND	FOUND	FOUND	NOT FOUND	NOT FOUND
A0A0J8B121	BVRB_016290	3	1	158	FOUND	FOUND	FOUND	FOUND	FOUND
A0A0J8B988	BVRB_5g123130	1	1	539	FOUND	NOT FOUND	NOT FOUND	FOUND	NOT FOUND
A0A0J8BHN7	BVRB_1g022890	2	2	489	FOUND	FOUND	FOUND	FOUND	FOUND
A0A0J8ERT5	BVRB_7g165900	2	2	171	FOUND	FOUND	FOUND	FOUND	FOUND
A0A0J8CVE2	BVRB_2g036670	2	2	154	FOUND	FOUND	FOUND	FOUND	FOUND
A0A0J8B3X6	BVRB_003990	3	3	370	FOUND	FOUND	FOUND	FOUND	FOUND
A0A0J8B6K6	BVRB_007350	1	1	295	FOUND	FOUND	FOUND	FOUND	FOUND
A0A0J8B3W3	BVRB_005890	1	1	683	FOUND	FOUND	FOUND	NOT FOUND	NOT FOUND
A0A0J8CQF1	BVRB_4g075880	1	1	286	FOUND	FOUND	NOT FOUND	NOT FOUND	NOT FOUND
A0A0J8BFX3	BVRB_3g067930	1	1	104	FOUND	FOUND	FOUND	FOUND	FOUND
A0A0J8B325	BVRB_008590	1	1	1540	FOUND	FOUND	FOUND	NOT FOUND	FOUND
A0A0J8B4H2	BVRB_015830	2	2	476	FOUND	FOUND	FOUND	FOUND	FOUND
A0A0J8B2K2	BVRB_010880	2	2	463	FOUND	FOUND	FOUND	FOUND	FOUND
A0A0J8EG55	BVRB_9g209140	1	1	339	FOUND	FOUND	FOUND	FOUND	FOUND
A0A0J8B6A0	BVRB_9g224420	2	2	54	FOUND	FOUND	FOUND	FOUND	FOUND
A0A0J8B1E7	BVRB_014810	1	1	71	FOUND	FOUND	NOT FOUND	FOUND	NOT FOUND
A0A0J8BAW6	BVRB_7g178940	2	2	368	FOUND	FOUND	FOUND	FOUND	FOUND
A0A0J8BDT0	BVRB_4g095620	2	2	343	FOUND	FOUND	NOT FOUND	FOUND	FOUND
A0A0J8CQH5	BVRB_4g076150	1	1	218	FOUND	FOUND	FOUND	FOUND	FOUND
A0A0J8B5Z7	BVRB_8g200850	2	2	233	FOUND	FOUND	FOUND	FOUND	FOUND
A0A0J8B8K0	BVRB_6g156330	1	1	107	FOUND	NOT FOUND	FOUND	FOUND	FOUND
A0A0J8BGI9	BVRB_2g047900	2	2	154	FOUND	FOUND	FOUND	FOUND	FOUND
Q39421		2	2	117	FOUND	FOUND	FOUND	NOT FOUND	FOUND
A0A0J8CD77	BVRB_5g105600	4	4	4316	FOUND	FOUND	FOUND	FOUND	FOUND
A0A0J8BEW1	BVRB_2g044740	2	2	234	FOUND	FOUND	FOUND	FOUND	FOUND

Table S1. Table of all identified proteins

A0A0J8BE80	BVRB_1g021810	2	2	637	FOUND	FOUND	FOUND	FOUND	NOT FOUND
A0A0J8DVG4	BVRB_014820	1	1	98	FOUND	FOUND	FOUND	FOUND	FOUND
A0A0J8B7X1	BVRB_002660	1	1	310	NOT FOUND	FOUND	NOT FOUND	FOUND	NOT FOUND
A0A023ZQ94	rpoC2	2	2	1369	FOUND	FOUND	FOUND	NOT FOUND	FOUND
A0A0J8B7I7	BVRB_003640	1	1	150	FOUND	NOT FOUND	FOUND	FOUND	FOUND
A0A0J8BAG2	BVRB_7g179960	1	1	117	FOUND	FOUND	NOT FOUND	NOT FOUND	FOUND
A0A0J8B2P1	BVRB_010500	1	1	95	NOT FOUND	NOT FOUND	NOT FOUND	FOUND	NOT FOUND
A0A0J8E1F4	BVRB_7g180220	3	1	78	NOT FOUND	NOT FOUND	FOUND	NOT FOUND	NOT FOUND
A0A0J8BBL3	BVRB_4g093940	2	2	142	NOT FOUND	FOUND	FOUND	FOUND	NOT FOUND
A0A0J8BHH1	BVRB_2g044570	1	1	546	FOUND	FOUND	NOT FOUND	NOT FOUND	NOT FOUND
A0A0J8BWI7	BVRB_7g165050	1	1	323	FOUND	FOUND	NOT FOUND	FOUND	FOUND
A0A0J8DYS4	BVRB_003030	2	2	541	FOUND	FOUND	FOUND	NOT FOUND	FOUND
A0A0J8E835	BVRB_2g046480	1	1	437	NOT FOUND	FOUND	NOT FOUND	FOUND	FOUND
A0A0J8DU34	BVRB_022590	3	1	285	NOT FOUND	FOUND	NOT FOUND	FOUND	NOT FOUND
A0A0J8E0J4	BVRB_8g201210	1	1	220	FOUND	FOUND	NOT FOUND	FOUND	FOUND
A0A023ZQ71	psbH	1	1	79	FOUND	NOT FOUND	FOUND	FOUND	FOUND
A0A0J8B3I2	BVRB_005710	1	1	106	FOUND	FOUND	NOT FOUND	NOT FOUND	NOT FOUND
Q9AWA3	H3	2	1	98	FOUND	FOUND	NOT FOUND	FOUND	FOUND
A0A0J8CS27	BVRB_3g065770	2	2	800	FOUND	FOUND	FOUND	FOUND	FOUND
A0A0J7YP66	BVRB_036990	1	1	113	FOUND	FOUND	FOUND	FOUND	FOUND
A0A023ZPQ8	rpoB	3	3	1070	FOUND	FOUND	FOUND	FOUND	FOUND
A0A0J8D1U1	BVRB_2g031520	2	2	320	FOUND	FOUND	FOUND	FOUND	FOUND
A0A0J7YM04	BVRB_017690	11	1	269	FOUND	FOUND	FOUND	NOT FOUND	FOUND
A0A0J8E3D3	BVRB_5g125430	1	1	394	FOUND	FOUND	NOT FOUND	FOUND	FOUND
A0A0J8B4C4	BVRB_016040	1	1	93	FOUND	FOUND	NOT FOUND	FOUND	FOUND
A0A0J8BDL7	BVRB_2g044480	1	1	139	FOUND	FOUND	FOUND	NOT FOUND	FOUND
A0A0J8B7Z1	BVRB_002240	1	1	361	FOUND	FOUND	FOUND	FOUND	FOUND
A0A0J8B6H8	BVRB_008600	2	2	558	FOUND	FOUND	FOUND	FOUND	FOUND
A0A0J8B694	BVRB_009390	2	2	676	FOUND	FOUND	FOUND	FOUND	FOUND
A0A0J8CFT9	BVRB_5g113750	2	2	376	FOUND	FOUND	FOUND	FOUND	FOUND
A0A0J8B1S3	BVRB_013490	1	1	286	NOT FOUND	FOUND	NOT FOUND	NOT FOUND	NOT FOUND
A0A0J8B891	BVRB_5g126500	3	3	877	FOUND	FOUND	FOUND	FOUND	FOUND
A0A0J8B9F4	BVRB_8g201480	2	2	445	FOUND	FOUND	FOUND	FOUND	FOUND
A0A0J8B3F0	BVRB_005990	1	1	269	NOT FOUND	NOT FOUND	NOT FOUND	FOUND	FOUND
A0A0J8CQ53	BVRB_3g055820	1	1	313	FOUND	FOUND	FOUND	NOT FOUND	NOT FOUND
A0A0J8BQS3	BVRB_9g206110	3	3	500	FOUND	NOT FOUND	FOUND	FOUND	FOUND
A0A0J8BBP8	BVRB_4g093500	1	1	434	NOT FOUND	NOT FOUND	FOUND	FOUND	FOUND
A0A0J8BCG5	BVRB_5g124060	2	2	322	FOUND	FOUND	FOUND	FOUND	FOUND
A0A0J8B521	BVRB_000380	3	3	370	FOUND	NOT FOUND	FOUND	FOUND	FOUND
A0A0J8E3M7	BVRB_5g124350	3	3	752	FOUND	FOUND	FOUND	FOUND	FOUND
Q9FPQ0	Ger171	1	1	208	FOUND	FOUND	FOUND	FOUND	FOUND
A0A0J8BBP3	BVRB_6g155720	1	1	281	NOT FOUND	FOUND	NOT FOUND	FOUND	FOUND
A0A0J8B9Y0	BVRB_5g123990	2	2	448	FOUND	FOUND	FOUND	FOUND	FOUND
A0A0J8BCT4	BVRB_5g124050	1	1	170	FOUND	FOUND	NOT FOUND	FOUND	NOT FOUND
A0A0J8B7X6	BVRB_004070	1	1	218	FOUND	FOUND	FOUND	FOUND	FOUND

Table S1. Table of all identified proteins

A0A0J8BHJ1	BVRB_2g044760	1	1	493	FOUND	FOUND	FOUND	FOUND	FOUND
A0A0J8BSP5	BVRB_8g184920	1	1	221	FOUND	FOUND	FOUND	NOT FOUND	NOT FOUND
A0A0J8B2P4	BVRB_008680	2	2	537	FOUND	NOT FOUND	FOUND	FOUND	FOUND
A0A0J8CGJ9	BVRB_4g091200	1	1	291	FOUND	FOUND	FOUND	FOUND	FOUND
A0A0J8BHI7	BVRB_2g044710	1	1	1182	FOUND	FOUND	FOUND	FOUND	FOUND
V5QQP3	Bv9_224260_gezu.t1	1	1	236	FOUND	FOUND	NOT FOUND	NOT FOUND	NOT FOUND
A0A0J8BF48	BVRB_3g070600	1	1	432	FOUND	FOUND	FOUND	FOUND	FOUND
A0A0J8BAT4	BVRB_7g179130	2	2	124	FOUND	FOUND	FOUND	NOT FOUND	FOUND
A0A0J8AWX3	BVRB_032280	1	1	143	FOUND	FOUND	FOUND	FOUND	FOUND
A0A0J8E3G7	BVRB_5g125040	1	1	333	FOUND	FOUND	NOT FOUND	NOT FOUND	NOT FOUND
A0A0J8B6V5	BVRB_006130	1	1	310	NOT FOUND	NOT FOUND	FOUND	NOT FOUND	NOT FOUND
A0A0J8CMT0	BVRB_3g065650	1	1	375	FOUND	FOUND	NOT FOUND	FOUND	NOT FOUND
A0A0J8BAM0	BVRB_7g179730	1	1	377	FOUND	FOUND	FOUND	FOUND	FOUND
A0A0J8B798	BVRB_004440	1	1	211	NOT FOUND	FOUND	FOUND	NOT FOUND	FOUND
A0A0J8DXK5	BVRB_007010	2	2	301	FOUND	FOUND	FOUND	FOUND	FOUND
A0A0J8CX13	BVRB_3g051100	1	1	185	FOUND	FOUND	NOT FOUND	FOUND	FOUND
A0A0J8DWZ6	BVRB_008780	1	1	534	FOUND	FOUND	FOUND	FOUND	FOUND
A0A0J8BGI3	BVRB_2g047360	1	1	797	FOUND	FOUND	FOUND	FOUND	FOUND
A0A0J8BH16	BVRB_2g044820	2	2	807	FOUND	FOUND	FOUND	FOUND	FOUND
A0A0N9LT50	rpoC1	2	2	677	FOUND	FOUND	FOUND	FOUND	FOUND
A0A0J8DWX1	BVRB_009060	2	2	362	NOT FOUND	NOT FOUND	NOT FOUND	FOUND	NOT FOUND
A0A0J8B6R9	BVRB_8g200730	1	1	94	FOUND	FOUND	NOT FOUND	FOUND	FOUND
A0A0J8B7U5	BVRB_003010	5	5	1390	NOT FOUND	NOT FOUND	NOT FOUND	FOUND	FOUND
A0A0J8BGU6	BVRB_2g046110	1	1	677	FOUND	FOUND	NOT FOUND	NOT FOUND	NOT FOUND
A0A0J8B956	BVRB_5g126620	1	1	603	FOUND	NOT FOUND	FOUND	FOUND	FOUND
A0A0J8BA12	BVRB_4g095380	1	1	321	NOT FOUND	FOUND	NOT FOUND	FOUND	NOT FOUND
A0A0J8B7N3	BVRB_004960	1	1	358	NOT FOUND	NOT FOUND	NOT FOUND	NOT FOUND	FOUND
A0A0J8BE12	BVRB_2g047490	1	1	167	NOT FOUND	FOUND	NOT FOUND	NOT FOUND	NOT FOUND
A0A0J8BDP3	BVRB_2g044720	1	1	154	NOT FOUND	FOUND	NOT FOUND	NOT FOUND	NOT FOUND
A0A0J8E465	BVRB_4g097560	4	4	653	FOUND	FOUND	FOUND	FOUND	FOUND
A0A0J8CZP6	BVRB_2g036730	1	1	334	FOUND	FOUND	FOUND	FOUND	NOT FOUND
A0A0J8B4G3	BVRB_002290	1	1	285	FOUND	FOUND	FOUND	FOUND	NOT FOUND
A0A411LD84	XTH1	1	1	285	FOUND	FOUND	FOUND	NOT FOUND	FOUND
A0A0J8B6I3	BVRB_008650	1	1	340	NOT FOUND	FOUND	NOT FOUND	NOT FOUND	FOUND
A0A0J8E120	BVRB_8g199830	1	1	639	FOUND	FOUND	FOUND	FOUND	FOUND
A0A0J7YLM1	BVRB_018790	1	1	406	FOUND	FOUND	FOUND	FOUND	FOUND
A0A0J8CIH9	BVRB_5g104540	2	2	374	FOUND	FOUND	FOUND	FOUND	NOT FOUND
A0A0J8B7N1	BVRB_003340	1	1	210	NOT FOUND	FOUND	NOT FOUND	NOT FOUND	NOT FOUND
A0A0J8B4A2	BVRB_004810 BVRB_004820	1	1	151	FOUND	FOUND	FOUND	NOT FOUND	FOUND
A0A0J8B7G9	BVRB_005530	1	1	571	FOUND	FOUND	NOT FOUND	FOUND	FOUND
A0A0J8CD57	BVRB_5g105660	2	2	888	FOUND	FOUND	FOUND	FOUND	FOUND
A0A0J8DX62	BVRB_008070	1	1	141	NOT FOUND	FOUND	FOUND	FOUND	FOUND
A0A0J8BE50	BVRB_4g093070	1	1	479	FOUND	FOUND	FOUND	FOUND	FOUND
A0A0J8BE84	BVRB_1g021800	1	1	405	FOUND	FOUND	NOT FOUND	NOT FOUND	NOT FOUND
A0A0J8CKG9	BVRB_4g075990	1	1	181	NOT FOUND	NOT FOUND	FOUND	FOUND	NOT FOUND

Table S1. Table of all identified proteins

A0A0J8C340	BVRB_6g145400	1	1	878	FOUND	FOUND	FOUND	FOUND	FOUND
A0A0J8BC20	BVRB_3g068070	3	3	292	FOUND	FOUND	FOUND	FOUND	FOUND
A0A0J8B5I1	BVRB_9g224870	1	1	312	NOT FOUND	NOT FOUND	FOUND	FOUND	FOUND
A0A0J8BKM8	BVRB_9g208980	1	1	948	FOUND	NOT FOUND	FOUND	FOUND	NOT FOUND
A0A0J8B3Y7	BVRB_003840	1	1	856	FOUND	FOUND	NOT FOUND	NOT FOUND	NOT FOUND
A0A0J8B9U4	BVRB_8g201280	1	1	155	FOUND	FOUND	NOT FOUND	FOUND	FOUND
A0A0J8B4U4	BVRB_014400	2	2	889	FOUND	FOUND	NOT FOUND	FOUND	FOUND
A0A0J8EHD4	BVRB_9g204770	1	1	195	FOUND	NOT FOUND	FOUND	NOT FOUND	FOUND
A0A0J8E2I3	BVRB_6g156300	1	1	239	FOUND	FOUND	FOUND	FOUND	NOT FOUND
A0A0J8BAU6	BVRB_7g179280	1	1	231	NOT FOUND	FOUND	FOUND	FOUND	FOUND
A0A7G2RM88	BVRB_8g200420	1	1	400	FOUND	FOUND	FOUND	FOUND	FOUND
A0A0J8B4G6	BVRB_004090	1	1	191	FOUND	FOUND	NOT FOUND	FOUND	FOUND
A0A0J8B8W0	BVRB_5g124090	1	1	217	NOT FOUND	FOUND	NOT FOUND	FOUND	FOUND
A0A0J8CV23	BVRB_2g036690	1	1	283	FOUND	NOT FOUND	NOT FOUND	NOT FOUND	FOUND
A0A0J8C066	BVRB_7g168050	1	1	598	FOUND	FOUND	FOUND	FOUND	FOUND
A0A0J8BPV2	BVRB_9g208970	1	1	230	FOUND	FOUND	FOUND	NOT FOUND	FOUND
A0A0J8B8V3	BVRB_6g155500	1	1	251	FOUND	NOT FOUND	FOUND	FOUND	FOUND
A0A0J8B8Y6	BVRB_000160	1	1	248	NOT FOUND	NOT FOUND	FOUND	FOUND	NOT FOUND
A0A0J8B716	BVRB_007120	1	1	1535	FOUND	FOUND	FOUND	FOUND	FOUND
A0A0J8FJ52	BVRB_3g056030	2	2	587	FOUND	FOUND	NOT FOUND	NOT FOUND	NOT FOUND
A0A0J8FHR1	BVRB_3g059810	1	1	270	FOUND	FOUND	NOT FOUND	NOT FOUND	FOUND
A0A0J8B6J6	BVRB_007530	1	1	816	NOT FOUND	FOUND	FOUND	FOUND	FOUND
A0A0J8CWI5	BVRB_2g033130	2	1	486	FOUND	FOUND	NOT FOUND	NOT FOUND	NOT FOUND
A0A0J8B4U5	BVRB_000900	1	1	308	FOUND	FOUND	FOUND	FOUND	FOUND
A0A0J8BVA5	BVRB_8g188100	17	2	1072	NOT FOUND	FOUND	FOUND	FOUND	FOUND
Q7XY04		1	1	112	FOUND	NOT FOUND	FOUND	NOT FOUND	FOUND
A0A0J8EXZ9	BVRB_6g143960	1	1	358	NOT FOUND	FOUND	NOT FOUND	FOUND	FOUND
A0A0J8B8W1	BVRB_9g225190	1	1	247	FOUND	NOT FOUND	FOUND	NOT FOUND	FOUND
A0A0J8B9I7	BVRB_5g125280	1	1	277	NOT FOUND	NOT FOUND	NOT FOUND	FOUND	NOT FOUND
A0A0J8BB58	BVRB_7g177790	1	1	2353	FOUND	FOUND	NOT FOUND	NOT FOUND	FOUND
A0A0J8BI59	BVRB_1g020710	1	1	790	NOT FOUND	NOT FOUND	NOT FOUND	NOT FOUND	FOUND
A0A0J8F7C9	BVRB_5g104420	2	2	2116	FOUND	FOUND	NOT FOUND	FOUND	FOUND
A0A0J8B7H3	BVRB_003960	1	1	478	FOUND	FOUND	NOT FOUND	FOUND	NOT FOUND
A0A0J8DYG5	BVRB_003790	1	1	1062	FOUND	NOT FOUND	FOUND	FOUND	FOUND
A0A0J8DYC3	BVRB_004150	1	1	341	FOUND	NOT FOUND	NOT FOUND	FOUND	NOT FOUND
A0A0J8B407	BVRB_003780	1	1	632	NOT FOUND	FOUND	NOT FOUND	NOT FOUND	NOT FOUND
J3S7K9		1	1	595	FOUND	FOUND	NOT FOUND	FOUND	FOUND
A0A0J8CKQ5	BVRB_4g090790	2	2	652	FOUND	FOUND	FOUND	FOUND	FOUND
A0A0J8B8W3	BVRB_6g155400	1	1	719	FOUND	NOT FOUND	NOT FOUND	FOUND	FOUND
A0A0J8B6B4	BVRB_8g199860	2	2	615	FOUND	NOT FOUND	NOT FOUND	NOT FOUND	FOUND
A0A0J8BB55	BVRB_3g070470	1	1	341	NOT FOUND	NOT FOUND	NOT FOUND	NOT FOUND	FOUND
A0A0J8CM03	BVRB_3g065260	1	1	299	NOT FOUND	NOT FOUND	FOUND	NOT FOUND	NOT FOUND
A0A0J8CVF6	BVRB_2g036810	1	1	348	NOT FOUND	FOUND	NOT FOUND	NOT FOUND	FOUND
A0A7G2RM13	BVRB_8g200380	1	1	1316	FOUND	FOUND	NOT FOUND	FOUND	FOUND
A0A0J8B7U4	BVRB_7g179270	1	1	331	FOUND	NOT FOUND	FOUND	FOUND	FOUND

Table S1. Table of all identified proteins

A0A0J8BDA1	BVRB_3g067700	1	1	347	NOT FOUND	NOT FOUND	FOUND	FOUND	NOT FOUND
A0A0J8B5S6	BVRB_011730	1	1	1760	NOT FOUND	NOT FOUND	FOUND	FOUND	FOUND
A0A0J8BB59	BVRB_3g070520	1	1	418	NOT FOUND	NOT FOUND	NOT FOUND	FOUND	FOUND
A0A0J8B8Y2	BVRB_5g124290	2	2	863	FOUND	FOUND	NOT FOUND	NOT FOUND	FOUND
A0A0J8B171	BVRB_015610	1	1	102	FOUND	FOUND	FOUND	FOUND	FOUND
A0A0J8CKM8	BVRB_4g090940	1	1	949	FOUND	FOUND	FOUND	FOUND	FOUND
A0A0J8B6L8	BVRB_007320	1	1	514	NOT FOUND	NOT FOUND	NOT FOUND	NOT FOUND	NOT FOUND
A0A0J8B871	BVRB_001830	1	1	353	FOUND	FOUND	FOUND	NOT FOUND	FOUND
A0A0J8DXR4	BVRB_006000	1	1	585	FOUND	NOT FOUND	NOT FOUND	NOT FOUND	FOUND
A0A0J8E6U8	BVRB_3g067260	1	1	336	NOT FOUND	FOUND	NOT FOUND	NOT FOUND	NOT FOUND
A0A0J8CX25	BVRB_2g031860	1	1	302	FOUND	FOUND	FOUND	FOUND	FOUND

Table S2. List of 569 proteins that differ significantly between samples and cluster locations of each protein in heat map

Protein IDs in Cluster 1	Protein IDS in Cluster 2	Protein IDS in Cluster 3	Protein IDS in Cluster 4	Protein IDS in Cluster 5	Protein IDS in Cluster 6
A0A0J8B555	A0A0J8B832	A0A0J8F9W0	A0A0J8CWI5	A0A0J8BH59	A0A0J8B2Q1
A0A7G2RLY6	A0A0J8BDP3	A0A0J8B7B7	A0A0J8B871	Q9MF87	A0A0J8B792
C7DYC4	A0A0J8CFT9	A0A0J8BPV2	A0A0J8CKM8	Q9MF62	Q9MF98
A0A0J8B3J3	A0A0J8B744	A0A0J8EYC2	A0A0J8B694	A0A0J8CMT0	A0A0J8B3J3
Q9MF98	A0A0J8B6Q9	A0A0J8B913	A0A0J8BE50	A0A0J7YMB8	A0A7G2RLY6
A0A0J8B792	A0A0J8B7I9	A0A0J8CFG6	A0A0J8DWD6	A0A0J8FB46	A0A0J8BF45
A0A0J8B8K1	A0A0J8B8Y7	A0A0J8B833	A0A0J8BC20	A0A0J8BA12	A0A0J7YP66
A0A0J8B6G4	A0A0J8E0H9	A0A0J8BF57	A0A0J8ER97	A0A0J8CKG9	A0A0J8B8V3
A0A0J8E8T6	A0A0J8DX62	A0A0J8BDA5	A0A0J8CNY8	A0A0J8B3F0	A0A023ZPQ8
A0A0J8B6B4	A0A0J8B6I3	A0A0J8B4Q2	A0A0J8CZN6	A0A0J8CU05	A0A0J8DY36
A0A0J8BBB6	A0A0J8CQ53	A0A0J8FQB8	A0A0J8B5V5	A0A0J8CV33	A0A0J8BGK6
A0A0J8BDS4	A0A0J8B131	A0A0J8B878	A0A0J8BDS6	A0A0J8AWX7	A0A0J8B956
A0A0J8B2P1	A0A0J8B4H2	A0A0J8E1V0	A0A0J8EMH9	A0A0J8BBJ2	A0A0N9LT50
A0A0J8CZN0	A0A0J8FHR1	A0A0J8E8M2	A0A0J8B9G9	A0A023ZR79	C7DYC4
A0A0J8DZ88	A0A0J8CNY8	A0A0J8BH02	A0A0J8DZT0	A0A0J8EGX4	I6PD11
A0A0J8C066	A0A0J8BI59	A0A0J8CKR1	A0A0J8BGL1	A0A0J8B5H5	A0A0J8B7Z1
A0A0J8B4N3	A0A0J8FG96	A0A023ZQU4	A0A023ZQV6	A0A0J8CD77	A0A0J8BBC3
A0A0J8BGI3	A0A0J8B5V5	A0A7G2RMM8	A0A0J8B8N6	A0A0J8BW11	A0A0J8BG04
A0A0J8B5I1	A0A0J8EMH9	A0A0J8B717	A0A0J8CVE2	A0A023ZQC6	A0A0J8B4N3
A0A0J8BB59	A0A0J8B767	A0A7G2RMG2	A0A0J8BFV0	A0A0J8E886	A0A0J8BGI3
A0A0J8CQH5	A0A0J8B2F5	A0A0J8BAE9	A0A0J8CTR3	A0A023ZPX6	A0A0J8C066
A0A0J8BGA9	A0A0J8BDF3	A0A0J8BLH6	A0A0J8B6B4	A0A0J8E6S4	A0A0J8DZ88
A0A0J8CI11	A0A0J8DZT0	A0A0J8CWJ9	A0A0J8B716	A0A0J8B936	A0A0J8CQH5
A0A023ZQ84	A0A0J8B9G9	A0A0J8BDL7	A0A0J8BH16	A0A0J8CS02	A0A0J8BGA9
A0A0J8B708	A0A0J8BGL1	A0A0J8BBP3	A0A0J8DZW6	A0A0J8BEG8	A0A0J8CI11
A0A0J8B6M1	A0A023ZQV6	A0A0J8E465	A0A0J8DX62	A0A0J8B7L7	A0A023ZQ84
A0A0J7YP66	A0A0J8CZN6	A0A0J8B4U4	A0A0J8B8Z8	A0A023ZPT1	A0A0J8B708
A0A0J8B8V3	A0A0J8B8N6	A0A0J8DXK5	A0A0J8CL57	O49812	A0A0J8B8Y6
A0A0J8B2P4	A0A0J8B293	A0A0J8ERT5	A0A0J8FG58	A0A0J8BBV5	A0A0J8FQE7
A0A0J8BCI1	A0A7G2RM13	A0A0J8CFS9	A0A0J8B6D7	A0A0J8B3I8	J3S7K9
A0A0J8B2K2	A0A0J8CWP6	A0A0J8BAT7	A0A0J8B417	A0A0J8BHP0	A0A0J8B8D4
A0A0J8EGU5	J3S7K8	A0A0J8DXV5	A0A0J8E4R6	A0A023ZRB4	A0A0N9LT71
A0A0J8DZ59	A0A0J8DWZ6	A0A0J8CFU5	A0A0J8FG96	A0A0J8CI46	A0A0J8B365
A0A0J8FEI6	A0A0J8BA63	A0A0J8CV90	A0A0J8BB86	Q9XFW7	A0A0J8B411
A0A0N9LTA0	A0A0J8DY30	A0A0J8CX02	A0A0J8C340	A0A0J8CRX9	A0A023ZPY0
A0A023ZQR1	A0A0J8CMT7	A0A0J8DZ23	A0A0J8B705	A0A023ZQ71	A0A023ZQH2
A0A0J8CFT3	A0A0J8B640	A0A0J8B9R6	A0A0J8BAP2	A0A0J8CS99	A0A0J8B981
A0A0J8B7T8	A0A0J8B891	A0A0J8CVA6	A0A0J8E6Y2	A0A0J8B6A0	A0A023ZQ47
A0A0J8BDJ6	A0A0J8B716	A0A0J8B8J2	A0A0J8E055	A0A0J8B4F5	A0A0J8BGJ7
A0A0J8B1C6	A0A0J8B3B5	A0A0J8BCX9	A0A0J8CVC3	A0A0J8B9A8	A0A0J8B9I7
A0A0J8BHE8	A0A0J8BCR7	A0A0J8BCV9	A0A0J8B4D2	A0A0J8B6J6	A0A023ZQW4
A0A0J8BQJ1	A0A0J8FC93	A0A0J8BGU6	A0A0J8B5A3	A0A0J8E3D3	A0A0J8CWV6

Table S2. List of 569 proteins that differ significantly between samples and cluster locations of each protein in heat map

A0A0J8B770	A0A0J8BCC1	A0A0J8DZL2	A0A0J8BEJ7	A0A0J8B4C4	A0A0J8CZN0
A0A023ZPS4	Q39421	A0A0J8BVB1	A0A0J8F9V0	A0A0J8CG74	A0A0J8B782
A0A0J8EMK8	A0A0J8B5Z4	A0A0J8B9R8	A0A0J8CIE0	A0A0J8B3A6	A0A0J8D0Z9
A0A0J8B3D2	A0A0J8B523	A0A0J8E8Y0	A0A0J8BHD9	A0A0J8B972	A0A0J8B2K2
A0A0J8BCI6	A0A0J8B8C4	A0A0J8DXQ5	A0A0J8CKI9	A0A0J8B454	A0A0J8EGU5
A0A0J8B227	A0A0J8B6K6	A0A0J8E6V5	A0A0J8CQK4	A0A0J8B8W3	A0A0J8DZ59
A0A0N9LT50	A0A0J8BSP5	A0A0J8B797	A0A0J8B787	A0A0J8CW97	A0A0J8FEI6
A0A0J8B956	A0A0J8B5Z7	A0A0J8B4A2	A0A0J8BPT2	A0A0J8BAA8	A0A0N9LTA0
I6PD11	A0A0J8EHD4	A0A0J8B382	A0A0J8CQI2	A0A0J8B4L3	A0A023ZQR1
A0A0J8BGK6	A0A0J8BAW6	A0A0J8E0B8	A0A0J8CM16	A0A0J8BEJ3	A0A0J8CFT3
A0A023ZPQ8	A0A0J8B661	A0A0J8E5W2	A0A0J8B2U4	A0A0J8B2H4	A0A0J8B7T8
Q0QG29	A0A0J8B6M7	A0A0J8B8W0	A0A0J8BBZ0	A0A0J8B402	A0A0J8BDJ6
Q5U6B5	A0A0J8B8A3	V5QQP3	A0A0J8BM46	A0A0J8B3K4	A0A0J8B1C6
A0A023ZR91	A0A0J8DZD4	A0A0J8ER90	A0A0J8C3V6	A0A0J8BDT0	A0A0J8BHE8
A0A023ZQS5	A0A0J8E2Y3	A0A0J8BAD6	A0A0J8CTT9	A0A0J8DX36	A0A0J8BQJ1
A0A023ZQD7	A0A0J8B3C9	A0A0J8B8R9	A0A1U9XPM6	A0A0J8DWP7	A0A0J8B770
A0A023ZQ99	A0A023ZQ52	A0A0J8B4W7	A0A0J8B999	A0A0J8DWR3	A0A023ZPS4
A0A023ZRF6	A0A0J8B489	A0A0J8DU34	A0A0J8BDA1	A0A0J8BD72	A0A0J8EMK8
A0A0J8E5V8	A0A0J8DXR4	A0A0J8B1E7	A0A0J8B1V3	A0A0J8B312	A0A0J8B3D2
Q39440	A0A0J8E1F4	A0A0J8CWI9	A0A0J8B2S4	A0A0J8B8E4	A0A0J8BCI6
Q9SPH6	A0A0J8B1Q4	A0A0J8CQF1	A0A0J8B5P2	A0A0J8DXP3	A0A0J8B227
A0A0J8ELP0	A0A0J8B3W3	A0A0J8ES22	A0A0J8BEJ9	V5QQV5	A0A0J8BBB6
A0A0J8EH07	A0A0J8B325	A0A0J8E3G7	A0A0J8BCW5	A0A0J8B9F4	A0A0J8BDS4
A0A0J8BB62	A0A023ZRD1	A0A0J8BWI7	A0A7G2RM29	A0A0J8FQT7	A0A0J8B5D6
A0A0J8BPV6	A0A023ZQ94	A0A0J8BCT4	A0A0J8B9C1	Q9AWA3	A0A0J8BB71
Q9MF86	A0A0J8BAM0	A0A0J8BEX1	A0A0J8BPU7	A0A0J7YLM1	A0A0J8BKL6
A0A0J8BQQ3	A0A0J8DYS4	A0A0J8B7R3	A0A0J8BEA4		A0A0J8B6M1
A0A0J8BD15	A0A0J8BVA5	A0A0J8BAG2	A0A0J8BC72		A0A0J8BCI1
A0A0J8BH75	Q9FPQ0	A0A0J8DVB5	A0A0J8BHS3		A0A0J8B2P4
A0A0J8F517	O81266	A0A0J8B5N7	A0A0J8BUP3		A0A0J8E8T6
A0A0J8B521	A0A0J8E380	A0A0J8C157	A0A0J8CM32		A0A0J8BE80
A0A0J8BHK5	A0A0J8B5D5	A0A0J8E0J4	A0A0J8BES3		A0A0J8B8K1
A0A023ZQB9	A0A0J8BAW1	A0A023ZQA3	A0A0J8FP32		Q9SM24
A0A0J8EGU0	A0A0J8B9C6	A0A0J8B6S1	A0A0J8E049		A0A0J8BBP8
A0A0J8DW49	A0A0J8B9H8		A0A0J8CWK4		A0A023ZR01
A0A0J8B9R3	A0A0J8CN27		A0A0J8CBM6		A0A0J8BQS3
A0A0J8CIG0	A0A0J8AWX3		A0A0J8BBN6		A0A0J8CKQ5
A0A023ZRF1			A0A0J8BF48		A0A0J8B3X6
A0A0J8FQE7			A0A0J8B5T2		A0A0J8B3G0
A0A0J8E6S4			A0A0J8B714		A0A023ZRF1
A0A0J8B411			A0A0J8B7X0		Q9MF79
A0A0J8BB71			A0A0J8B8N3		A0A023ZRF6
A0A0J8BKL6			A0A0J8BHI7		Q0QG29
A0A0J8CRX9			A0A0J8BDD0		Q5U6B5
A0A0J8CU05			A0A0J8DXP0		A0A023ZR91

Table S2. List of 569 proteins that differ significantly between samples and cluster locations of each protein in heat map

A0A0J8E886	A0A0J8B6I6	A0A023ZQS5
A0A023ZPT1	A0A0J8E9M3	A0A023ZQD7
O49812	A0A0J8CMT7	A0A023ZQ99
A0A0J8BBV5	A0A0J8B5J0	A0A023ZQB9
A0A0J8B3I8	A0A0J8B6R9	Q9MFD6
A0A0J8BEG8	A0A0J8FJ52	A0A0J8E998
A0A0J8B5S6	A0A0J8B4H2	A0A023ZQ82
A0A0J8B782	A0A0J8BSP5	A0A0N9LU76
A0A0J8B936	A0A0J8B3Y7	A0A0J8E5V8
A0A0J8D0Z9	A0A0J8BKM8	Q39440
A0A023ZPY0	A0A0J8B3I2	Q9SPH6
A0A023ZQH2	A0A0J8B1S3	A0A0J8BB62
A0A0J8B981	A0A0J8FHR1	A0A0J8BPV6
A0A023ZQ47	A0A0J8BE12	Q9MF86
A0A0J8BGJ7	A0A0J8BDP3	A0A0J8BQQ3
A0A0J8B8D4	A0A0J8EG55	A0A0J8BD15
A0A0N9LT71	A0A0J8E2I3	A0A0J8BH75
A0A0J8B365	A0A0J8BE84	A0A0J8F517
A0A023ZQW4	A0A0J8CQ53	A0A0J8DW49
A0A0J8CWV6	A0A0J8DVI3	A0A0J8B9R3
A0A023ZPX6	A0A0J8B4G3	A0A0J8CIG0
J3S7K9	A0A0J8B648	A0A0J8ELP0
A0A0J8B7I7	A0A0J8B2V7	A0A0J8B521
A0A0J8CV23	A0A0J8B2V2	A0A0J8EH07
A0A0J8B7U5	A0A0J8DVD8	Q0QG26
A0A0J8CS99	A0A0J8BCR7	A0A0J8B7I7
A0A0J8B6A0	A0A0J8FC93	A0A023ZQ96
A0A0J8B4F5	A0A0J8D1C1	Q9MF46
Q9MF46	A0A0J8E0F9	A0A0J8B7U5
A0A0J8B9A8	A0A0J8FEF7	A0A0J8EGU0
A0A0J8CKQ5	A0A0J8BLI2	A0A0J8BHK5
A0A0J8B2Q1	A0A0J8B8Q3	A0A0J8D1H6
Q0QG26	A0A0J8D0B1	A0A0J8B6G4
Q9MFD6	A0A0J8CGH0	A0A0J8B555
Q9MF79	A0A0J8B4F7	
A0A0J8E998	A0A0J8E360	
A0A023ZQ82	A0A0J8BHG7	
A0A0N9LU76	A0A0J8E4J4	
A0A023ZQ96	A0A7G2RM13	
A0A0J8DY36	A0A0J8CWP6	
A0A0J8CV33	A0A0J8B5L8	
Q9SM24	A0A0J8B293	
A0A0J8B7Z1	A0A0J8DYK7	
A0A0J8B5D6	A0A0J8B593	
A0A0J8BBP8	A0A0J8CFT9	

Table S2. List of 569 proteins that differ significantly between samples and cluster locations of each protein in heat map

A0A023ZR01	A0A0J8B4G6
A0A0J8BBC3	A0A0J8E4V4
A0A0J8B3X6	A0A0J8E0H9
A0A0J8B3G0	A0A0J8B375
A0A0J8BQS3	A0A0J8CWM8
A0A0J8DXP3	A0A0J8CS27
	A0A0J8B7X6
	A0A0J8EH11
	A0A0J8F7C9
	A0A0J8CWJ3
	A0A0J8E0P8
	A0A0J8B4D7
	A0A0J8B6R4
	A0A0J8BBQ5
	A0A0J8E1N5
	A0A0J8B2D3
	A0A0J8BC28
	A0A0J8BM33
	A0A0J8BEC0
	A0A0J8E8U1
	A0A0J8BHN8
	A0A0J8BHH1
	A0A0J8B7X1
	A0A0J8CS33
	A0A0J8B407
	A0A0J8CQA6
	A0A0J8CBR1
	A0A0J8CGJ9
	A0A0J8E3S1
	A0A0J8CL67
	A0A0J8E1T2
	A0A0J8E2P5
	A0A0J8B7W0
	A0A0J8B2B2
	A0A0J8B6L3
	A0A0J8BGI9
	A0A0J8DZ93
	A0A0J8B8U9
	A0A0J8B9J6
	A0A0J8CX13
	A0A0J8EXZ5
	A0A0J8B9B6
	A0A0J8BD51
	A0A0J8E2K8
	A0A0J8B525

Table S2. List of 569 proteins that differ significantly between samples and cluster locations of each protein in heat map

A0A0J8BDJ3
A0A0J8BH48
A0A0J8BD59
A0A0J8BKN7
A0A0J8F6Z5
A0A0J8B2R5
A0A0J8BET8
A0A0J8CV41
A0A0J8CW87
A0A0J8B9V6
A0A0J8E325
A0A0J8BHE5
A0A0J8BD81
A0A023ZQE9
A0A0J8B7I9
A0A0J8B2X7
A0A0J8BHR2
A0A0J8E6N8
A0A0J8BZW3
A0A0J8BH96
A0A0J8B5T5
A0A0J8C0U2
A0A0J8FG78
A0A0J8B6M2
A0A0J8E591
A0A0J8BE74
A0A0J8B3T7
A0A0J8E0G2
A0A0J8CB59
A0A0J8CQL5
A0A0J8BW84
A0A0J8CWD5
A0A0J8CZP6
A0A0J8BCG5
A0A0J8B352

Figure S3. PCA Analysis Without HC

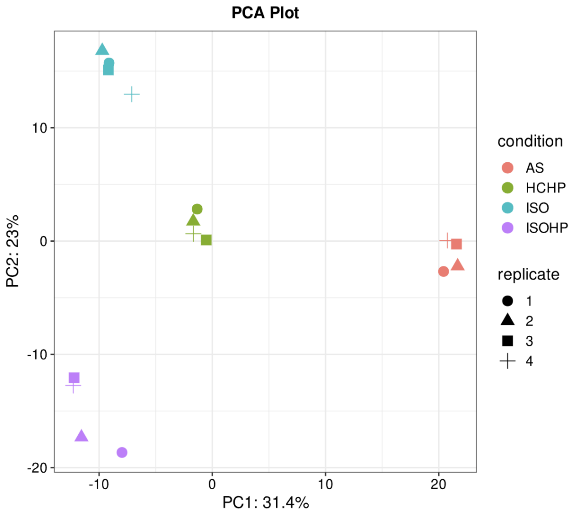

Supplement: Supplementary file 1 — jf2c09190_si_001.pdf [file jf2c09190_si_001.pdf]
